# Follicular dendritic cells in health and disease

**DOI:** 10.3389/fimmu.2012.00292

**Published:** 2012-09-21

**Authors:** Mohey Eldin M. El Shikh, Costantino Pitzalis

**Affiliations:** Centre for Experimental Medicine and Rheumatology, William Harvey Research Institute, Barts and the London School of Medicine and Dentistry, Queen Mary University of LondonLondon, UK

**Keywords:** follicular dendritic cell (FDC), antigen presentation, B-cells, germinal center reaction, T-cell-independent, Fc receptors, complement, autoimmunity

## Abstract

Follicular dendritic cells (FDCs) are unique immune cells that contribute to the regulation of humoral immune responses. These cells are located in the B-cell follicles of secondary lymphoid tissues where they trap and retain antigens (Ags) in the form of highly immunogenic immune complexes (ICs) consisting of Ag plus specific antibody (Ab) and/or complement proteins. FDCs multimerize Ags and present them polyvalently to B-cells in periodically arranged arrays that extensively crosslink the B-cell receptors for Ag (BCRs). FDC-FcγRIIB mediates IC periodicity, and FDC-Ag presentation combined with other soluble and membrane bound signals contributed by FDCs, like FDC-BAFF, -IL-6, and -C4bBP, are essential for the induction of the germinal center (GC) reaction, the maintenance of serological memory, and the remarkable ability of FDC-Ags to induce specific Ab responses in the absence of cognate T-cell help. On the other hand, FDCs play a negative role in several disease conditions including chronic inflammatory diseases, autoimmune diseases, HIV/AIDS, prion diseases, and follicular lymphomas. Compared to other accessory immune cells, FDCs have received little attention, and their functions have not been fully elucidated. This review gives an overview of FDC structure, and recapitulates our current knowledge on the immunoregulatory functions of FDCs in health and disease. A better understanding of FDCs should permit better regulation of Ab responses to suit the therapeutic manipulation of regulated and dysregulated immune responses.

## Introduction

In 1968, Andras K. Szakal and Michael G. Hanna, Jr. (Szakal and Hanna, Jr., 1968) from the Oak Ridge National Laboratory, Tennessee, USA; and Gustav J. Nossal et al. (Nossal et al., 1968) from the Walter and Eliza Hall Institute in Melbourne, Australia, published the first electron micrographs and description of FDCs. A number of names have been used, but a committee on nomenclature recommended the name “Follicular Dendritic Cell” and the abbreviation “FDC,” and this has been generally adopted (Tew et al., 1982).

The characteristics that distinguish FDCs from other cells of the immune system are their ability to retain antigen (Ag)-antibody (Ab)-complement complexes (i.e., immune complexes, ICs) long-term on their surfaces and their follicular localization. Unlike other immune accessory cells, FDCs lack phagocytic activity, lysosomes, lysozyme, and Birbeck granules. FDCs localized to the follicles of secondary lymphoid tissues form interactive networks or reticula of non-mobile Ag-bearing cells. Immobile FDCs in these FDC-reticula engage the mobile B and T-cells and other mobile cells trafficking through the follicles (Szakal et al., 1985; El Shikh et al., 2010).

**Figure 1:**
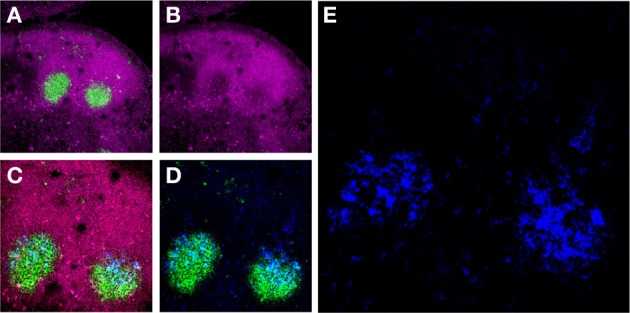
**Murine FDCs in lymph node (LN) GCs**. **(A)** Activated GC B-cells labeled with the mAb GL7 (green) are surrounded by a mantle of CD45Rhi (magenta) resting B-cells in two adjacent secondary B-cell follicles in the draining LNs of an immunized mouse. **(B)** These GC B-cells downregulate CD45R expression upon activation. **(C** and **D)** The activated GC B-cells are intimately associated with FDC Ag retaining reticulum (blue). **(E)** High magnification of the FDC reticulum retaining AMCA-conjugated ovalbumin (blue).

**Figure 2:**
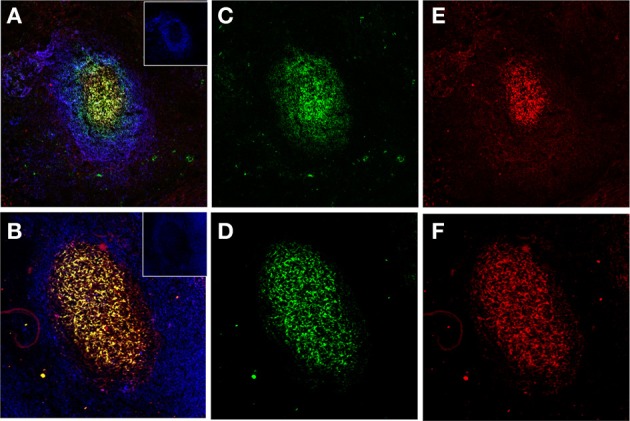
**Human FDCs in tonsillar sections trapping IgG/C3-opsonized Ags in ICs**. **(A** and **B)** Two B-cell follicles surrounded by resting IgD+ mantle B-cells (blue, insets in the right upper corner). The overlay of FDCs in the follicles (labeled green with CNA.42 mAb) and the FDC-trapped ICs (labeled red with anti-human IgG in the upper panel and anti-C3 in the lower panel) is orange-yellow. **(C** and **D)** FDCs labeled green with the FDC-specific mAb CNA.42. **(E)** Human IgG in the FDC-trapped ICs labeled red with anti-human IgG. **(F)** Human C3 in FDC-trapped ICs labeled red with anti-human C3.

FDCs in germinal center (GC) light zones of B-cell follicles (Figures 1 and 2) retain ICs via the complement receptors (CR1/CR2—CD35/CD21), the low affinity immunoglobulin gamma Fc region receptor II-B (FcγRIIB/CD32) and the low affinity immunoglobulin epsilon Fc region receptor II (FcεRII/CD23). In addition, FDC dendrites bear complement 3 (C3) fragments (iC3b, C3d, and C3dg) attached to the ICs and adjacent cell membranes via ester linkages at the FDC-B-cell interface. Engagement of BCRs with FDC-retained Ags is critical for GC development, B-cell survival, Ig class switching, production of B memory cells, somatic hypermutation (SHM), selection of somatically mutated B-cells with high affinity receptors, affinity maturation, induction of secondary Ab responses, and regulation of serum IgG and IgE levels. Moreover, FDCs exert a pivotal role in organizing the lymphoid micro-architecture, and abrogation of FDC networks as a consequence of ablation of Lymphotoxin (LT) trimers and Tumor Necrosis Factor-α (TNF-α), either through homologous recombination or by pharmacological means, abolishes the sophisticated lymphoid architecture compartmentalization (Kosco-Vilbois, 2003; Allen and Cyster, 2008; El Shikh et al., 2009b, 2010).

FDCs multimerize intact monomeric proteins and present Ags in ICs to B-cells with regular periodicity *in vivo* and *in vitro*. ICs trapped via FDC-FcγRIIB in the absence of complement are displayed in a periodic manner with 200–500 Å spacing between epitopes (Szakal et al., 1985; Sukumar et al., 2006b, 2008). In contrast to proteolysis and the MHC-restricted peptide presentation by DCs to T-cells, and similar to T-cell-independent (TI) type 2 Ags and higher order protein arrays, FDCs display native monomeric proteins as multimerized ICs with repeating immunogenic epitopes thus allowing extensive BCR cross-linking and induction of rapid B-cell activation, proliferation, GC formation, and Ig secretion to T-cell-dependent (TD) Ags in a TI manner. In addition to this FcγRIIB-dependent Ag presentation, FDC-derived B-cell Activating Factor of the TNF family (BAFF) and C4b-binding protein (C4bBP) are critical for TI B-cell activation (El Shikh et al., 2009a, 2010).

Engagement of BCRs by free Ag can induce B-cell activation; however, an immunogen is converted into ICs as soon as Ab is produced in a primary response, and instantaneously in recall responses by Ab persisting from previous immunizations. IgG-ICs coligate BCR with FcγRIIB and inhibit B-cell activation via the tyrosine-based inhibitory motif (ITIM). In contrast, IgG-ICs trapped by FDC-FcγRIIB do not engage B-cell-FcγRIIB and consequently ITIM-mediated signaling is minimized (Tew et al., 1976, 2001; Qin et al., 2000; Aydar et al., 2003, 2004b; El Shikh et al., 2006).

Not only FDCs provide the necessary Ag for B-cell stimulation but they orchestrate the removal of self-Ags released from apoptotic cells in the GC environment. FDCs secrete the bridging factor Mfge8, which crosslinks apoptotic cells in the GCs and the phagocytic “tingible body macrophages” (TBMs). This promotes selective debris removal and engulfment of dead cells. Mice lacking LT or LT receptors, which are devoid of FDCs, or lacking the bridging protein Mfge8 itself, are significantly susceptible to autoimmunity suggesting the role of FDCs in protection against autoimmunity by expediting the removal of potentially self-reactive debris from the GCs (Kranich et al., 2008).

Beyond their immunoregulatory functions, FDCs are critically involved in the pathogenesis of several diseases. FDCs are important HIV reservoir where FDC-trapped HIV persists for long periods and can reignite infection and perpetuate the disease (Smith et al., 2001). Moreover, FDCs are the essential sites of prion replication in lymphoid tissues (McCulloch et al., 2011), and they provide a microenvironment that supports B-cell lymphomas and establish a protective sanctuary for malignant cells from otherwise effective drugs (Lee et al., 2012).

In addition, several lines of evidences support the critical role of FDCs in the pathogenesis of autoimmune diseases. Chronic inflammatory autoimmune disorders frequently display follicles with IC-bearing FDCs, autoreactive GCs, and ongoing affinity maturation (Aloisi and Pujol-Borrell, 2006; Manzo et al., 2010). Moreover, interference with FDC reticula attenuates autoreactive GC formation, reduces pathogenic auto-Ab titers and memory B lymphocytes and ameliorates arthritis (Gray and Skarvall, 1988; Gray, 1993; Barrington et al., 2002; Anolik et al., 2008; Victoratos and Kollias, 2009; Vinuesa et al., 2009; Manzo et al., 2010).

In this review we address the current knowledge about the structure and function of FDCs and their role in immune regulation in health and disease.

## FDC morphology, development, Ag retention, and isolation

### FDC morphology

Light and electron microscopy studies of FDCs have revealed structural details that help clarify FDC functions. FDCs are slightly larger than lymphocytes and possess many fine dendritic processes. These dendrites extend and intimately interact with neighboring cells, creating a unique microenvironment. This intimate interaction with surrounding lymphocytes appears to be important for FDCs to provide potent signals that promote humoral immune responses. Scanning electron microscopy (SEM) has identified two morphological types of FDCs: one having filiform or finger-like processes, and one with “beaded” dendrites. The transition from one form of FDC into another appears to be related to the formation of a specialized Ag delivery system. The resulting “beads” are called “iccosomes” to denote that they consist of immune complex-coated bodies (Schnizlein et al., 1985; Szakal et al., 1985; Sukumar et al., 2006b, 2008; El Shikh et al., 2007a).

FDCs possess a scanty cytoplasm with few mitochondria, rough endoplasmic reticulum, a Golgi apparatus, and vesicles. They have irregular, sometimes bilobed, euchromatic nuclei containing distinct nucleoli. The dendritic processes of FDCs appear in two general forms: some are attenuated, with folds and intermittent thickenings that form a variety of differently shaped cytoplasmic extensions, while others form more uniform, highly convoluted, labyrinthine configurations. These dendritic processes interdigitate with one another and form a vast network or reticulum on which ICs are trapped (Schnizlein et al., 1985; Szakal et al., 1985; Sukumar et al., 2006b, 2008; El Shikh et al., 2007a).

### FDC origin, development, and lymphoid tissue organization

The cellular origin of FDCs is still controversial and various cell types have been proposed as a possible origin of FDCs. FDCs, at least in their mature form, are stationary, however, the possibility of an inflow of earlier progenitors of FDCs from other sites could not be fully excluded (Kasajima-Akatsuka and Maeda, 2006).

At present there are data supporting a haematopoietic origin and more data supporting a stromal cell origin. FDCs are radiation resistant making it difficult to study development using adoptive transfer models. Information available on FDCs is based primarily on studies using humans and rodents. However, FDCs are present and functional in birds and if FDCs are defined broadly as cells with the ability to trap ICs, they appear to exist in all jawed vertebrates, including amphibians, reptiles, and fish (El Shikh et al., 2009b).

Using mice homozygous for the SCID mutation, which lack T, B lymphocytes, and FDCs, demonstrated that after reconstitution with bone marrow from donor mice, the FDCs of the reconstituted mice expressed the donor phenotype. These authors concluded that FDC precursors came from bone marrow (Kapasi et al., 1993a,b, 1994; Szakal et al., 1995a,b; Kapasi et al., 1998).

On the other hand, stromal cells expressing FDC markers have been described in several independent studies, and seem to be present in the splenic white pulp before the development of functionally and ultrastructurally recognizable FDC. Using an *in silico* subtraction approach, gene expression of FDCs was determined and compared with that of follicular stromal cells microdissected from the spleen of SCID mice and a remarkably close relationship in gene expression patterns was found (Wilke et al., 2010). However, one of the major limitations in the study of FDC origin is the paucity in markers specific for the various stages of FDC maturation that would allow for discriminating FDC precursors from B-cells as well as from other stromal cells (Aguzzi and Krautler, 2010; Wilke et al., 2010).

Recent transcriptome analysis showed that FDCs express many mesenchyme-associated genes suggesting that FDCs are specialized mesenchymal cell population within the GCs of lymphoid tissues (Mabbott et al., 2011). It was also suggested that cytokines from lymphocytes and macrophages involved in inflammatory process may be responsible for differentiating stromal cells into a FDC phenotype (Cho et al., 2012a).

Another recent study has suggested a mechanism of FDC development that involves both resident and migratory cells. Specifically, it was proposed that a FDC is generated by a cell fusion event between a stromal cell and a migratory CD35^+^B220^+^ precursor cell, which is consistent with several observations of binucleate FDCs (Murakami et al., 2007; Allen and Cyster, 2008). Moreover, differentiation of FDCs as a specialized form of myofibroblasts that derive from bone marrow stromal cell progenitors has been also suggested (Munoz-Fernandez et al., 2006; Sipos and Muzes, 2011).

TNF and the related molecule LT are essential for FDC development, and mice deficient in these cytokines, their receptors, or associated downstream signaling molecules fail to properly develop FDCs and GCs in secondary lymphoid organs. Through irradiation chimera and adoptive transfer experiments, it was established that TNF and LT were required on lymphocytes, specifically B-cells for normal FDC development. The differential role of soluble and membrane bound TNF in FDC development has been also investigated with more significant role of soluble TNF in FDC development in primary follicles and the membrane-bound TNF form in FDCs of the GCs (Allen and Cyster, 2008; Tumanov et al., 2010).

FDCs help maintain primary follicles as a B-cell exclusive niche and they act to retain and promote the survival of GC B-cells within GCs. Within two days of FDC ablation, primary B-cell follicles lose their homogeneity and become disorganized bands of cells around T zones. Ablation of FDCs during the GC response causes rapid GC B-cell dispersal, death, and disappearance of the GCs (Wang et al., 2011).

The cardinal feature of FDCs is the surface retention of native Ags for extended periods of time and presentation of these Ags together with costimulatory signals to B-cells during normal and abnormal immune responses. This unique property of Ag retention and presentation by FDCs: (1) occurs in different sites of secondary lymphoid tissues, including the spleen, LNs, and mucosa-associated lymphoid tissues (MALT); (2) can be induced in tertiary lymphoid tissues in different organs as a consequence of chronic inflammatory and autoimmune reactions; (3) depends on Ag retention on FDCs, which is directly associated with different Ag transport mechanisms in secondary and tertiary lymphoid tissues; (4) can be induced in cells of haematopoietic and stromal origin under physiological and pathological conditions *in vivo* and *in vitro*. Consequently, the origin of FDCs may be differentially regulated in different anatomical sites, under different sets of microenvironmental conditions, and by the cells and routes that deliver the Ag to the FDC reticulum. Our knowledge is still expanding in the field of FDC biology and more needs to be investigated in the processes of FDC differentiation and the mechanisms of Ag transport to their reticula in different anatomical locations before a final conclusion can be drawn on their origin.

### Ag transport to the FDC reticulum

A notable feature of the FDC network is that it is located centrally in the follicle and typically does not extend to the subcapsular sinus (SCS), interfollicular regions or T-cell zone. The logic for this separation of FDCs from the sites of earliest Ag capture has not been defined but it prompted studies on the mechanisms of Ag transport to the FDC reticulum dating back to the early 1980s.

Perhaps being centred in the follicle and not in substantial contact with macrophages, DCs, or circulatory fluids provide a protected environment in which opsonized Ags can be displayed for long periods without being proteolysed or removed by phagocytic cells. However, the physical isolation of FDCs in follicles necessitates mechanisms for Ag to travel from the first point of capture to the FDC (Cyster, 2010).

In the early 1980s, elegant light and electron microscopic studies indicated that as early as 1 min after Ag injection in passively immune mice, localization of Ag occurs at distinct sites in the SCS and subjacent areas of the cortex on the afferent side. At these sites, between 1 min and 24 h, the Ag forms light microscopically identifiable trails which reaches progressively deeper into the cortex with time toward individual follicular regions. By 24 h this apparent migration of Ag is complete, and the Ag is localized in follicles. Electron microscopy indicated that the apparent migration of ICs was mediated by a group of cells observed in the migration path that had ICs sequestered on their surface or in plasma membrane infoldings. These Ag transporting cells (ATC) were relatively large non-phagocytic cells, with lobated or irregular euchromatic nuclei and cell processes of various complexities. ATCs observed in or near the SCS appeared to be less differentiated, whereas those located deeper in the cortex, appeared more differentiated, interdigitated with Ag-retaining dendritic cells, and shared morphologic characteristics with FDC (Szakal et al., 1983).

More recently, it was reported that large MW proteins (>60 kDa) injected into passively immune mice form ICs that activate complement, resulting in covalent attachment of C3 (C3-IC). SCS macrophages (SCSM) have a low rate of Ag internalization and degradation (Cyster, 2010), and the C3-coated ICs are shuttled via the SCSM-CR3 onto naïve B-cell-CR1/2-CD32 that migrate to the FDC reticulum and off-load the ICs to the FDC-CR1/2-CD32. In the absence of pre-existing specific Abs, innate recognition proteins such as natural IgM; C-type lectins such as MBL and ficolins; pentraxins, including C-reactive protein; and other complement activators could effectively bind the foreign protein and activate complement, then the C3-coated ICs follow the SCSM-CR3/naïve B-cell-CR1/2-CD32/FDC-CR1/2-CD32 transport mechanism. C3-coated smaller MW proteins access the B-cell follicles via the follicular conduits that intersect with FDCs, providing a direct connection for C3-coated Ags to bind to the FDC surface via CR1/2-CD32. DCs residing in the LN medulla may also capture C3-opsonized Ags and hand off the complexes to FDCs in the B-cell follicle (Gonzalez et al., 2011).

It has been also reported that SCSM themselves transport ICs directly to FDCs after immunization with Ag in adjuvant (Cyster, 2010), and the strategic positioning of FDCs around follicular conduits in the B-cell follicles of the LNs gives them direct access to capture soluble Ags draining from subcutaneous sites even in the absence of Ag-specific Abs (Bajenoff and Germain, 2009).

Both marginal zone (MZ) B and B-1-cells help reorganization of FDC networks (Ferguson et al., 2004; Lopes-Carvalho et al., 2005) and B-1-cells promote the development of FDCs reticula (Wen et al., 2005). The continuous shuttling of MZ-B-cells to B-cell follicles has been recently documented and the potential interaction between MZ-B-cells and Ags in ICs on FDCs has been proposed (Cinamon et al., 2008; MacLennan, 2008). In the spleen, MZ-B-cells that express relatively high levels of CD21 and CD35 pick up C3-ICs from the sinus and deliver the complexes to FDCs as they migrate though the follicles (Cinamon et al., 2008; MacLennan, 2008; Gonzalez et al., 2011). Natural and specific Abs differentially regulates Ag transport in the spleen. Particulate proteins expressed on virus-like particles were transported efficiently to murine splenic FDCs in the absence of prior immunity via natural IgM Abs and complement, whereas soluble proteins required Ag-specific IgM or IgG Abs (Link et al., 2012).

### FDC isolation and *in vitro* GC reactions

Major challenges delayed the systematic analysis of FDC functions in health and disease. Retrieval and characterization of FDC-retained Ags, isolation of the Ag-retaining FDCs, detection of picogram quantities of Ags in GCs, and the lack of *in vitro* models for GC reactions represented major technical difficulties in investigating the biology of FDCs. The current development of FDC isolation techniques with >90% purity (Sukumar et al., 2006b), and methods for setting up GC reactions *in vitro* (El Shikh et al., 2007b, 2009a; Wu et al., 2008, 2009); where the influence of Ags, ICs, FDC-, T-cell-, and B-cell-regulatory molecules can be manipulated at will; provide a pathway to analyse the molecular interaction between the cellular and molecular components of the GC reaction. Based on the lack of CD45 expression, a recent study described a novel method for FDC isolation from the spleen of naive mice by flow cytometry. The isolated FDCs, which accounted for ~0.2% of the spleen cells of naive mice, were CD45^−^, FDC-M2^+^, and ICAM-1^+^, and supported the survival and LPS-induced proliferation of B-cells via FDC-BAFF-dependent pathway (Usui et al., 2012).

FDCs are fragile cells and are tightly associated with B-cells which hamper the process of FDC isolation. Several FDC lines were established to overcome these problems, however, as these cells are maintained over several weeks in culture, their phenotype no longer reflects the *in vivo* situation (Aguzzi and Krautler, 2010; Wilke et al., 2010). FDCs require constitutive LT-R-mediated stimulation from surrounding lymphocytes to maintain their maturation status. In the absence of this stimulation FDC rapidly dedifferentiate (Mabbott et al., 2011).

Artificial engineering of lymphoid tissue equivalents is an emerging field that aims to provide models for therapeutic applications of vaccines, and drugs used in the treatment of chronic inflammation, autoimmunity, and cancer. Being the hotspot of Ab production, more work on developing *in vitro* GC reactions is critical for these artificial lymphoid tissues (Tan and Watanabe, 2010).

## T-cell-dependent (TD) and independent (TI) antigens, the germinal center reaction (GCR), and the immunoregulatory functions of FDCs

FDCs critically regulate Ag presentation to B-cells in the GCR. In the following section we will introduce the types of Ags and the GCR before we discuss the specific immunoregulatory functions of the FDCs.

### TD and TI Ags

Long before T and B lymphocytes were discovered, it was known that certain Ags of bacterial origin were fundamentally different from protein Ags. Lipopolysaccharide (LPS), for example, was found to be immunogenic at very low concentrations and even 1000 molecules could induce a detectable immune response dominated by 19S Abs (IgM) (Moller, 1965; Landy and Baker, 1966).

With regard to their capacity for Ab-induction, Ags are classified as T-cell dependent (TD) or T-cell independent (TI). In the immune responses to TD Ags, provision of cognate T-cell help to B lymphocytes is essential for B-cell proliferation, immunoglobulin (Ig) secretion, class switch recombination (CSR), affinity maturation, and memory B-cell generation. On the contrary, TI Ags induce B-cell activation and Ab production without T-cell help. TI Ags are further divided into TI type 1 Ags (TI-1), which are polyclonal B lymphocyte activators, and TI type 2 (TI-2) Ags which are classically polysaccharides fit for extensive crosslinking of multiple BCRs.

Protein Ags without repetitive epitopes require cognate T-cell help to induce high affinity B-cell immunity. These TD Ags are processed and presented by Ag presenting cells (APCs) which activate Ag-specific helper T (Th) cells in the T-cell zones of secondary lymphoid tissue. Activated Th cells expand and differentiate into effector Th cells that regulate the development of Ag primed B-cells. Under Th cell control, these primed B-cells can switch Ig isotype, terminally commit to the plasma cell pathway or enter the GCR to experience cycles of SHM, affinity maturation, selection of high affinity B-cells, and memory B-cell generation. Ag-specific Th cells contribute to the GCRs where they critically regulate the affinity-based selection of memory B-cells. A long-lived memory Th cell compartment is established in the immune responses to TD Ags, where Ag rechallenge induces exaggerated cellular expansion in the memory B and T-cell compartments (Parker, 1993; McHeyzer-Williams et al., 2003; Jeurissen et al., 2004).

A complex mixture of TD Ags (peptides and proteins) and TI Ags (glycolipids and complex polysaccharides) are present in microorganisms (Leyva-Cobian et al., 1997). Pathogen-associated TI-1 Ags are polyclonal B-cell activators most of them are derived from components of the cell membranes, the cytosol, or excretion/secretion products of bacteria, viruses, and parasites. The Abs secreted by B-cells stimulated with polyclonal activators are non-specific and recognize heterologous as well as homologous Ags. The molecular mechanisms triggered by polyclonal activators derived from microorganisms have not been elucidated completely; however, several lines of evidences support that these molecules are able to activate B-cells through TLRs. For example, DNA or oligodeoxynucleotides containing CpG motifs [CpG-oligodeoxynucleotide (ODN)] act via the intracellular receptor TLR-9. Conversely, LPS in complexes with the LPS-binding protein (LPSBP) is captured by CD14 which then associates with the TLR-4-myeloid differentiation protein 2 complex on B-cells. This initiates signaling pathways, which involve MyD88, and also leads to the activation of NF-κ B (Baumgarth, 2000; Montes et al., 2007). Induction of TI GCRs by certain pathogen-associated Ags can promote expansion of B-cell clones expressing V regions selected over evolutionary time to recognize common multivalent structures on the surface of such pathogens. Because SHM does not take place efficiently in TI GCRs, the specificity of the selected V regions would not be altered, and such a process would not pose a threat to self tolerance (Manser, 2004).

TI-2 Ags are classically large MW polysaccharides displaying repeating immunogenic epitopes with poor biodegradability, and complement fixation ability. These Ags can crosslink BCRs and stimulate Ab production by mature B-cell in athymic nude mice lacking MHC class II restricted T-cell help [detailed in (Dintzis et al., 1976, 1982; Brunswick et al., 1988, 1992; Mond et al., 1995; Sulzer and Perelson, 1997; de Vinuesa et al., 2000; Vos et al., 2000; Lentz and Manser, 2001; Zubler, 2001)].

In spite of their chemical diversity, structural analysis of classic TI-2 Ags has shown that all share a minimal MW of 100 kDa and repetitive epitopes that are expressed on a stable backbone with a two-dimensional epitope spacing of 95–675 Å. An extended length of the backbone of 460 nm, carrying 48 epitopes engaging 10–50 mIg receptors forming a small number of highly crosslinked clusters of mIg molecules with 14-fold reduction in the diffusion coefficient of the bound mIg receptors is critical for delivering the BCR-mediated signal in TI-2 responses (Vos et al., 2000).

In view of recent studies, the operational characteristics of TI-2 Ags have been extended beyond classic polysaccharides. Reports on the requirements for generation of Ab responses to repetitive determinants on polymers and higher order structures such as viral capsid proteins indicated that high MW arrays of Ag are efficient in eliciting Ab responses independent of T-cell help, whereas their less ordered counterparts are less immunogenic and require T-cell help (Dintzis et al., 1983; Bachmann and Zinkernagel, 1997). Data from several viral models including, Coxsackie, influenza, foot-and-mouth disease, vesicular stomatitis, and Sindbis viruses, indicate that virus-specific IgM responses are mounted when athymic nude, TCRα - TCRβ - TCRαβ - and TCRαβγδ-knockout mice are infected. All of the antiviral TI Ab responses reported so far are directed to viral Ags repetitively displayed in the virions (Szomolanyi-Tsuda and Welsh, 1998). Moreover, Abs bound to strictly ordered, but not to irregularly arranged, viral Ags dramatically enhanced the induction of anti-Abs after a single immunization and without using adjuvants (Fehr et al., 1997). Similar to TI-2 Ags, and higher order protein arrays on viral capsids, the ability of FDCs to retain ICs in a periodic manner allows multimerization of monomeric proteins that generally express only a single copy of each antigenic determinant and facilitates the multivalent presentation of these determinants in an array fit for crosslinking of multiple BCRs and induction of Ab responses in the absence of T-cell help (El Shikh et al., 2009a, 2010).

A clinically important group among the TI-2 Ags are the bacterial capsular polysaccharides. Capsular polysaccharides of *Streptococcus pneumoniae*, *Haemophilus influenzae*, and *Neisseria meningitidis* are responsible for the bacterial virulence; and Abs to capsular polysaccharides provides protection against invasive infections with these bacteria. Maturation of the immune response to TI-2 Ags in humans is delayed. The delay in Ab formation to encapsulated bacteria renders infants and young children highly susceptible to infections with encapsulated bacteria, especially from the age of 4 to 6 months on, when the placenta-derived maternal IgG is metabolized. Therefore, children younger than 2 years of age are more at risk for invasive infections caused by encapsulated microorganisms (Jeurissen et al., 2004).

TI B-cell activation is further discussed in the immunoregulatory functions of FDCs.

### The GCR

GCs are highly structured tissue microdomains in activated B-cell follicles wherein B lymphocytes undergo clonal expansion, CSR, Ab gene diversification, and affinity maturation.

GCs were first observed in 1884 by Flemming and his associates who concluded that GCs were one of the major sources of lymphocytes throughout the body. Though the name given to the sites of intense proliferation in the follicles is still used, we now know that the conclusion that the cells originate/germinate within the GC is not true. The term “reaction” in GCR is used to stress the reactive nature of the GC response, i.e., the development (and decline) of a GC as a result of antigenic stimulation (Nieuwenhuis and Opstelten, 1984).

GCRs differ slightly from organ to organ and from species to species. GCs also form in atypical sites (referred to as tertiary lymphoid tissues) such as in the stomach during *Helicobacter pylori* infections, the eye during Chlamydia infections, blood vessels in atherosclerosis, joints in rheumatoid arthritis, the thyroid in Hashimoto's thyroiditis, and the lung in the chronic inflammatory diseases of the respiratory system (Kosco-Vilbois et al., 1997). GC formation is restricted to homoiothermic animals. Associated with the ability to produce GCs in their lymphoid tissues, birds, and mammals produce Abs of heterogeneous affinity, increasing in the secondary as compared to the primary response. The characteristic fast rise of serum Ab after a secondary injection of Ag may provide a survival advantage in the defence against pathogens that multiply more rapidly at the higher body temperature (Thorbecke et al., 1994; Manser, 2004). Furthermore, aging is associated with defective GCRs associated with abnormal functions of the GC cellular components (Kosco et al., 1989; Szakal et al., 1990, 1992, 2002; Smith et al., 1991; Tew et al., 1993; Thorbecke et al., 1994; Zheng et al., 1997; Aydar et al., 2002, 2003, 2004a).

GCs arise in primary lymphoid follicles of secondary lym-phoid tissues. The sites that become involved are those receiving the antigenic stimulus via drainage of lymph, blood, or specialized cells in the epithelium called “M” cells. The GCR is a complex process involving numerous cellular and cell surface components together with multiple signaling pathways (Nieuwenhuis and Opstelten, 1984; MacLennan, 1994; Kosco-Vilbois et al., 1997; Cozine et al., 2005; Vinuesa and Cyster, 2011).

The primary B-cell follicle contains a mixture of recirculating naïve and memory B-cells. Upon B-cell activation in the T-cell zone, a few committed Ag specific B-cells migrate into the follicle and expand clonally. Several studies indicated that low numbers of Ag-specific B-cells can be seen within the follicles in the primary response as early as 36 h post-immunization. Three to four Ag-specific B-cells colonize a follicle and go through repeated rapid cell cycles increasing some 4000-fold in around three days. In doing this, they fill the spaces between the network of FDCs. Within 72–84 h of exponential growth the cells differentiate to form a mature GC chracterized by the presence of a light zone and a dark zone. The proliferating cells move to one pole of the GC and become centroblasts. Centroblasts produce non-proliferating centrocytes that are thought to migrate to the light zone of the GC, which is rich in Ag-trapping FDCs and T follicular helper (Tfh) cells. Small, non-cycling follicular B-cells are then moved closer together, forming the follicular mantle. The light zone contains a rich network of FDCs that have the capacity to take up Ag and hold it on their surface for periods of more than a year. The Ag is held as an IC in a native unprocessed form; but the Ag may be taken up from FDC by B-cells, which can process and present it to T-cells. The open structure of GCs allows high-affinity Ag-specific B-cells to be recruited to an ongoing GCR. This enhances competition for the FDC-bound Ags and ensures that rare high-affinity B-cells can participate in Ab responses (MacLennan, 1994; Kosco-Vilbois et al., 1997; MacLennan et al., 2000; McHeyzer-Williams, 2003; Natkunam, 2007; Schwickert et al., 2007).

One of the hallmarks of the GCR is the introduction of somatic mutations into IgV genes of GC centroblasts, mainly single base pair mutations, followed by an efficient selection procedure that ensures selection of Abs with higher affinity (Song et al., 1998; McHeyzer-Williams et al., 2000; Chan and Brink, 2012). SHM adds as many as one mutation per cell per division which provides a substrate for selection of higher affinity and more diverse populations of cells. SHM can result in increased affinity of the BCR for Ag, a critical event in affinity maturation and the production of high-affinity Abs. Conversely, SHM can cause loss of affinity, or altered specificity with the acquisition of autoreactivity. Centroblasts continually give rise to non-proliferating centrocytes, which enter the FDC network where they are subjected to selection. The selection is based on the ability of a centrocyte first to bind Ag held on FDC, and second to present this in a processed form to CD4 T-effector cells, which are sited toward the outer edge of the FDC network. Cells that fail to undergo positive selection die by apoptosis, the default pathway for centrocytes that do not receive selection signals. Selection eliminates those cells that have lost Ag-binding activity, or have acquired autoreactivity (MacLennan et al., 2000; McHeyzer-Williams, 2003; Tarlinton, 2008; Vinuesa and Cyster, 2011; Shlomchik and Weisel, 2012b).

Upon exiting the GCR, selected B-cells can differentiate into two long-lived compartments; memory B-cells, and bone marrow-resident plasma cells. Both cell populations participate in sustaining high-titred Ab levels. Memory B-cells recirculate in the periphery, where they can survive for decades in humans. Upon re-encounter with the Ag for which they are specific, memory B-cells can rapidly proliferate and differentiate into plasma cells secreting high affinity Abs. on the other hand, bone marrow-resident long-lived plasma cells secrete Abs for long periods of time (MacLennan et al., 2000; McHeyzer-Williams, 2003; Kallies et al., 2004; Tarlinton, 2008; Elgueta et al., 2010; Good-Jacobson and Shlomchik, 2010; Vinuesa and Cyster, 2011; McHeyzer-Williams et al., 2012; Peperzak et al., 2012; Shlomchik and Weisel, 2012a).

GCs persist for about 3 weeks following immunization (MacLennan, 1994), however, in mice immunized with protein Ag in adjuvant (Shlomchik and Weisel, 2012b) and TI GC responses to multimerized protein Ags on FDCs (El Shikh et al., 2009a) the GC response can persist detectably for more that 8 weeks. Moreover, immunization with viruses induces long-term GCs associated with proliferating Ag-specific B-cells (Tarlinton, 1998) and continuous influx of T-cells (Vinuesa et al., 2010). With the demise of the GCR, the dendritic processes of the FDC form tightly folded convolutions of membrane where the Ag is retained extracellularly forming depots of FDC-retained Ags that may contribute to the recall responses (Sukumar et al., 2008; El Shikh et al., 2010).

The contribution of T-cells to the GCR has been studied extensively as T-cell help to B-cells is a fundamental aspect of adaptive immunity and the generation of immunological memory. Tfh cells differentiate in response to immunization or infection and provide help to GC B-cells. Tfh cells, initially described in 2000–2001, are CD4^+^ T-cells that mature in a Bcl-6-dependent manner and are capable of providing IL-21-dependent help to B-cells for GC formation and switched Ag-specific Ab responses (Vinuesa and Cyster, 2011). This population is characterized by a core set of factors essential for their generation and function; including Bcl-6, IL-21, and CXCR5 expression; CD28 costimulation; ICOS ligation; and SAP-mediated interactions with cognate B-cells (Fazilleau et al., 2009; Crotty, 2011; Nutt and Tarlinton, 2011; Linterman et al., 2012; Weinstein et al., 2012).

The original description of GCs entailed definite T-cell dependency (Nieuwenhuis and Opstelten, 1984), however, recent studies indicated that high-affinity B-cells can be induced to form large GCs in response to (4-hydroxy-3-nitrophenyl) acetyl (NP)-Ficoll in the absence of T-cells (de Vinuesa et al., 2000; Lentz and Manser, 2001). Moreover, FDC-retained TD Ags induce TI B-cell activation and Ig secretion in responses costimulated by FDC-BAFF and C4bBP (El Shikh et al., 2009a). Therefore, T-cells may be required for latter stages of the GCR, but they are dispensable for the induction and initial development of this response (Lentz and Manser, 2001).

Several models have been proposed for the cellular dynamics during the GCR (McHeyzer-Williams et al., 2000; McHeyzer-Williams and McHeyzer-Williams, 2005; Allen et al., 2007; Or-Guil et al., 2007), and although there is no typical GCs in terms of size, recent studies indicated that GCs have typical cellular ratios attained during the established phase of the response (Wittenbrink et al., 2011).

Autoreactive and malignant GC B-cells induce a unique class of disorders because they originate from cells of the immune system that divert from the normal maturation programmes, via genetic rearrangements or somatic mutations. As SHM and gene rearrangements are the physiological landmarks of Ag-driven GC responses, the risk for genetic lesions (and hence autoimmunity and malignant transformation) is exponentially enhanced (Hollowood and Goodlad, 1998; Guzman-Rojas et al., 2002; Vinuesa et al., 2009; Manzo et al., 2010). GC B-cells with low Ag affinity and autoreactivity are eliminated via apoptosis and are rapidly cleared by TBMs. Inefficient clearance of apoptotic cells results in autoimmunity that is thought to be mediated by various intracellular molecules possessing danger-associated molecular patterns (DAMPs), including nuclear self-Ags. DAMPs can be released from apoptotic cells undergoing secondary necrosis due to disruption of the apoptotic cell clearance programmes within the GCs (Rahman, 2011).

Although the enzyme activation-induced deaminase (AID) is essential for creating Ab diversification by causing mutations during the GCR, it also greatly enhances the chance of B-cell lymphoma development. AID increases the mutation rate to one per thousand bases, six orders of magnitude more than spontaneous mutagenesis, and can ultimately lead to mutations of proto-oncogenes and chromosomal translocations resulting in a wide variety of B-cell lymphomas that are thought to derive from GC B-cells based on their carrying somatically mutated V region genes (Peperzak et al., 2012; Shlomchik and Weisel, 2012b).

In tertiary lymphoid tissues, infiltrating B- and T-lymphocytes organize themselves into ectopic follicles and GCs. This has been observed in several autoimmune, chronic inflammatory, and neoplastic diseases. Ectopic GCs are architecturally similar to GCs in conventional secondary lymphoid organs, and comprise proliferating B-cells and networks of FDCs. T-cells are also a regular component of ectopic GCs where they provide cognate T-cell help required for the progression of the GC B-cell response (Aloisi and Pujol-Borrell, 2006; Carragher et al., 2008).

Although some elements of the cellular interactions between cell types within GCs have now been visualized, it is yet to attain the ability to image over extended time periods in order to have clearer image of the GC kinetics.

### The immunoregulatory functions of FDCs

#### Ag presentation

Studies of TD Ags including ovalbumin, horseradish peroxidase, and human serum albumin indicated that these immunogens are almost instantaneously converted into ICs by Abs persisting from prior immunization(s). Moreover, in primary responses, ICs form as soon as Ab is produced and these ICs are trapped in the FDC reticula. Ag trapping and retention is exquisitely localized to draining lymphoid tissues. Ag injection into a single limb of an immune mouse will be localized to the draining LNs and to a lesser extent in the spleen (Mandel et al., 1980; Donaldson et al., 1986). With time, the Ag persisting on FDCs becomes more and more focused to the lymphoid follicles nearest the site of Ag injection and by 1 year, specific Ab forming cells are almost exclusively confined to the most proximal LN (Donaldson et al., 1986). Remarkably, once Ags are trapped on FDCs, they remain relatively unaffected by a variety of manipulations including gamma-irradiation, stress, and treatment with a number of anticancer drugs. However, cortisone acetate injections result in the loss of a significant portion of persisting Ag although the reason for this remains unclear (El Shikh et al., 2009b).

FDCs trap ICs via Fc and C receptors (Tew et al., 2001; El Shikh et al., 2006). In the spleen, Ag retention requires the presence of complement (Klaus and Humphrey, 1977; Tew et al., 1979), whereas, in the draining LNs, FDCs are capable of trapping ICs in the absence of complement (Tew et al., 1979; El Shikh et al., 2006). Complement-mediated IC trapping in the draining lymph nodes of FcγRIIB^−^/^−^ mice is normal compared to WT controls although retention of ICs over time is reduced in FcγRIIB^−^/^−^ mice suggesting FcγRIIB is important in long-term maintenance of the ICs (Tew et al., 1976). Remarkably, engagement of FDC-FcγRIIB with ICs plays a critical role in activating FDCs. Binding of ICs to FDC-FcγRIIB induces FDC activation which leads to dramatic upregulation of FDC-ICAM-1, -VCAM-1 and -FcγRIIB itself (El Shikh et al., 2006). In addition, FDC-FcγRIIB plays a major role in B-cell activation with Ag-specific and anti-BCR ligation where the high density of FcγRIIB on FDCs binds Ig-Fc in the IC and consequently the ITIM signal delivered via co-ligating BCR and B-cell-FcγRIIB is minimized (Aydar et al., 2003, 2004b).

FDCs multimerize native Ags in a periodic manner as initially discovered using SEM with Ags trapped *in vivo*. Szakal et al. reported patterns of orderly, spiraling, arrangement of ICs made up of alternating light and dark bands (Szakal et al., 1985), and recent studied of ICs trapping by FDCs *in vitro* confirmed Ag periodicity (Sukumar et al., 2008) and indicated that ICs are arranged on FDCs with a 200–500Å spacing between epitopes which correlates well with spacing optimal for BCR crosslinking and activation by TI type 2 Ags (Dintzis et al., 1976, 1983). The periodically arranged ICs interact and “zip” processes together which may help explain IC preservation and long-term retention (Sukumar et al., 2008).

Using two-photon microscopy, it was recently shown that the B-cell encounter with FDC-associated Ag could be detected for >1 week after immunization. B-cell-FDC contact times were often brief but occasionally persisted for >30 min and B-cells sometimes acquired Ag together with FDC surface proteins. These observations establish that FDCs serve as sites of B-cell Ag capture, with their prolonged display time ensuring that even rare B-cells have the chance of Ag encounter (Suzuki et al., 2009).

Persisting Ags on FDCs are critical for SHM which is important for affinity maturation. SHM is a late GC event beginning 9–11 days after primary challenge, and a late specific Ag signal (a week after priming) is delivered by Ags persisting on FDCs to promote SHM and affinity maturation (Wu et al., 2008). Moreover, Ags retained on FDCs are delivered to B-cells in the form of iccosomes, which are highly immunogenic. Iccosomes are readily endocytosed by GC B-cells, which process the Ag and present it to T-cells (Szakal et al., 1988).

Strikingly, FDCs display Ags even in the presence of high levels of specific Ab which might be expected to mask epitopes and thus block successful Ag presentation. However, B-cells cluster with FDCs forming a synapse at the point of FDC-B-cell contact and Ag-specific B-cells recognize FDC-Ag via their BCRs in the synapse even in the presence of high levels of Abs during the GC reaction (El Shikh et al., 2009b).

#### B-cell costimulation

***FDC-derived complement cosignals.*** In addition to presentation of multimerized Ags in arrays that extensively crosslink BCRs, FDCs provide costimultory signals that regulate Ig secretion in TD and TI Ab responses. FDC-CD21L, -C4bBP, -BAFF, and -IL-6 are major FDC-molecules known to signal B-cells. The important interaction between a complement-derived CD21 ligand on FDCs and CD21 on B-cells in the initiation of IgG responses has been well verified (Qin et al., 1998; Tew et al., 2001; Aydar et al., 2005). Coligation of BCR and CD21 facilitates association of BCR and B-cell co-receptor complex promoting CD19 phosphorylation by a tyrosine kinase associated with BCR (Carter et al., 1997). This cosignal dramatically augments stimulation delivered by engagement of BCR by Ag. Blockade of the CR2 ligand on FDCs by the use of soluble CR2 or use of B-cells from CR2 knockout mice (or B-cells with CR2 blocked) reduced Ab responses 10–1000 folds (Qin et al., 1998; Tew et al., 2001; Aydar et al., 2005). FDCs from C3 knockout mice, which cannot generate the CR2-binding fragments (iC3b, C3d, and C3dg), were unable to provide costimulatory activity (Qin et al., 1998; Fakher et al., 2001).

Not only do FDCs provide C3 (CD21L) as a complement derived cosignal, but FDC-C4bBP has been recently reported to engage B-cell CD40 and serve as a cosignal in FDC-dependent TI responses to polysaccharides. TI polysaccharide Ags fix the alternative complement pathway directly and the classical pathway indirectly by making ICs with specific IgM produced early in the response. In addition to accessory signals delivered by the complement derived CD21L, activated forms of both C3 (C3b) and C4 (C4b) bind C4 binding protein (C4BP), which in humans and likely in rodents, colocalizes with ICs on FDCs. C4bBP in ICs trapped on FDCs has been shown to signal B-cells via intact CD40 in short-lived Ag-specific TI GCs independent of T-cell derived CD40L (CD154). More specifically, in mice with normal FDCs, injection of a blocking mAb, FDC-M2, which recognizes an epitope on C4 bound within ICs, also inhibited TI GC development. C3- and C4-deficient mice showed impaired TI Ab responses consistent with the hypothesis that ICs on FDCs can provide Ag-specific, complement-derived, and CD40-mediated signals to B-cells initiating B-cell proliferation in TI GCs (Gaspal et al., 2006).

***FDC-derived cytokines.*** FDCs produce BAFF (Hase et al., 2004; Magliozzi et al., 2004; Zhang et al., 2005; El Shikh et al., 2009a; Manzo et al., 2010) that has the ability to support TI B-cell activation (Shulga-Morskaya et al., 2004; Groom et al., 2007). FDC-BAFF is critical for FDC-dependent B-cell activation (El Shikh et al., 2009a), and BAFF is directly involved in rescue, activation, and follicular homing of B-cells (Varin et al., 2010). BAFF binds to BAFF-R, TACI (transmembrane activator and calcium-modulator and cyclophilin ligand [CAML] interactor) and, BCMA (B-cell maturation Ag). The functional outcome of BAFF signaling is multifaceted and different receptors mediate different functions. Peripheral B-cell survival, plasma cell survival, MZ-B-cell integrity, GC maintenance, CD21 and CD23 expression, TI B-cell responses and Ig class switching all have been attributed to BAFF (Schneider, 2005).

Recent studies indicated a critical role for IL-6, whose primary source in GCs is FDCs (Kopf et al., 1998), in specific IgG responses and SHM (Wu et al., 2009). IL-6, directly or via IL-17, regulates B-cell activity (Deng et al., 2002), and blocking IL-6 from FDCs inhibits both IgG responses and SHM (Wu et al., 2009). FDC-BAFF, -IL-6, and -IL-15 can induce T-cell IL-4, IL-5, IFNγ, and TNF-α (Mori et al., 1996; Heijink et al., 2002; Huard et al., 2004; Park et al., 2004), and these cytokines can indirectly promote B-cell activation and Ig class switching.

***FDC-B-cell synapse.*** The integrity of the first BCR-mediated signal depends on extensive crosslinking of BCRs with IC-bearing FDCs which likely entails formation of a properly functioning FDC-B-cell synapse. FDC-CXCL-13 attracts follicular B-cells, and in the spleen MZ-B-cells, and helps organize follicles (Cyster et al., 2000; Estes et al., 2004). Within the follicles, the adhesion molecules ICAM-1 and VCAM-1 are critical for FDC-B-cell synapse formation and activation, and their blockade inhibits FDC-B-cell clustering and B-cell activation (Kosco et al., 1992; Maeda et al., 1995).

#### T independent B-cell activation

TI type 2 Ags trapped on FDCs can induce GCs in animals lacking cognate T and B-cell interactions. When mice with mutations that inactivate the *TCR Cβ* and *Cδ* genes were immunized with 4-hydroxy-3-nitrophenylacetyl (NP)-Ficoll, GCs with peanut agglutinin-binding B-cells were observed in the splenic follicles. These GCs contained mature IC-bearing FDCs and although they are rapidly induced, their duration is short (de Vinuesa et al., 2000; Lentz and Manser, 2001). Nevertheless, the finding that natural and synthetic multivalent Ags can induce Ig secretion in the absence of T-cells does not mean that T-cells do not play a part in TI responses and the involvement of several T-cell cytokines has been established (Vos et al., 2000).

Typically ~48 h is needed before primed T-cells are able to provide cognate help and it is unlikely that cognate T helper cells are involved in 48 h TI responses. It is important to appreciate that IC activated FDCs produce BAFF and IL-6 and that these cytokines can in turn rapidly induce cytokine production by T-cells (Huard et al., 2001, 2004; Heijink et al., 2002). Owing to their rapid intense cytokine production and their ability to provide B-cell help in a non-MHC-restricted fashion, γδ and NK T-cells could help in the early phases of TI responses. IFN-γ-mediated enhancement of Ig secretion has been demonstrated by *in vitro* addition of γδ T-cell clones to anti-δ-dex stimulated B-cells. Moreover, several reports indicate that pre-incubation of purified B-cells with IL-4, a rapid product of activated NK T-cells, prior to anti-δ-dex stimulation resulted in a 10-fold increase in Ig secretion within 6 h. It has been also shown that proliferation of B-cells stimulated with slg-dependent (anti-μ-dex) or -independent (LPS) polyclonal activators is markedly augmented *in vitro* by addition of purified FDCs in a dose-dependent fashion and cultures containing B-cells from athymic nude mice proliferated normally in the presence of anti-μ -dex plus rlL-4, implying that IL-4 provides adequate help (Burton et al., 1993).

The ability of FDCs to convert TD Ags into TI Ags was recently reported. TD Ags in ICs on FDCs are spaced 200–500 Å apart on the flexible backbone of FDC membranes which is geometrically fit for extensive BCR crosslinking. Moreover, FDCs provide BAFF and C4bBP, which are known to support TI B-cell activation. Nude or athymic mice challenged with ICs produce specific IgM in 48 hr while challenge with free Ag in adjuvant failed to induce IgM even after many weeks. Moreover, the draining lymph nodes of IC-challenged nude mice exhibit well-developed GCs associated with FDC Ag retaining reticula and plasmablasts within 48 h. IgM is the first class of Abs produced during primary Ab responses, and the ability to induce FDC-dependent, TI IgM responses may be critical in host defence in the initial phases of infections before T-cell help is provided or in disease conditions where T-cell insufficiency prevails (El Shikh et al., 2009a, 2010).

#### Major FDC regulatory and signaling molecules

Table 1 summarizes the currently characterized FDC regulatory and signaling molecules.

**Table 1:** **FDC regulatory and signaling molecules**.

**FDC molecule**	**Function**
BAFF	FDC-BAFF supports TI B-cell activation (El Shikh et al., 2009a), and is required for the proper formation of FDC networks within the GCs (Moisini and Davidson, 2009).
CD21/35 (CR1/2)	CRs are crucial for IC retention especially in the spleen (Tew et al., 1979; Qin et al., 1997, 1998; Carroll, 1998; Barrington et al., 2002). Genetic CD21 deficiency in humans adds to the molecular defects observed in human subjects with hypogammaglobulinemia and severe reduction in memory B-cells (Thiel et al., 2012).
CD21L (iC3b, C3d or C3dg)	Engagement of CD21 in the B-cell co-receptor complex by complement derived FDC-CD21L delivers a critical co-signal. Coligation of BCR and CD21 facilitates association of the two receptors and the cytoplasmic tail of CD19 is phosphorylated by a tyrosine kinase associated with the BCR complex. This co-signal augments stimulation delivered by Ag and blockade of FDC-CD21L reduces B-cell proliferation, activation induced cytidine deaminase, and Ab production 10–1000 fold (Croix et al., 1996; Fischer et al., 1996; Carroll, 1998; Qin et al., 1998; El Shikh et al., 2010).
CD23 (Fcε RII)	FDC-Fcε RII mediates IC retention, and the regulation of the GC reaction and IgE levels (El Shikh et al., 2009b; Gibb et al., 2010; Chaimowitz et al., 2011).
CD29, CD44	FDCs express CD44 and CD29 and FDC binding to collagen type I *in vitro* induces the regeneration of FDC processes and networks. CD44 also enhances B-cell adherence to FDCs allowing delivery of the FDC-derived B-cell survival signals including 8D6 and BAFF (El Shikh et al., 2007a).
CD32 (FcγRIIB)	FDC-FcγRIIB is critically involved in conversion of poorly immunogenic ICs into a highly immunogenic form, FDC activation, IC periodicity, long-term IC retention, and regulation of serum IgG levels (El Shikh et al., 2009b, 2010).
CD320 (8D6)	The 8D6 molecule inhibits apoptosis and influences both proliferation and Ab secretion by GC B-cells. Moreover, GC B-cells that are induced to differentiate into pre-plasma cells are the most sensitive to the neutralizing effects of anti-8D6 (Zhang et al., 2001; Li et al., 2004; Cho et al., 2008).
CD40	FDCs express CD40 and when incubated with either CD40L trimer or agonistic anti-CD40 Ab, the expression of FDC-CD23 is increased both at the mRNA and protein levels. FDC-CD23 helps regulate IgE levels (Payet-Jamroz et al., 2001; Sukumar et al., 2006a; Gibb et al., 2010).
CD54 (ICAM-1), CD106 (VCAM-1), MadCAM-1	Abs reactive with murine ICAM-1 and/or leukocyte functional Ag-I (LFA-I) interfere with FDC-B-cell clustering resulting in reduced B-cell proliferation. In addition, VLA-4 and VCAM-1 have been observed in GCs and likely also play a role in FDC-B-cell interactions. These adhesion molecules are thought to stabilize the FDC-B-cell synapse and promote interaction of FDC-Ag and FDC-costimulatory molecules with B-cells (Kosco et al., 1992; Maeda et al., 1995; Vinuesa et al., 2010).
CXCL13	CXCL13 is secreted by FDCs and acts as chemoattractant for B-cells via the CXCR5 chemokine receptor. FDC development and expression of this chemokine depend on LTα β, and TNF-α. The maintenance of lymphoid follicle structure is mediated by a positive feedback loop: CXCL13 stimulates B-cells to express high levels of LTα β, and TNF-α which stimulates FDCs to produce CXCL13 (Ansel et al., 2000; Cyster et al., 2000; Manzo et al., 2005; Wang et al., 2011).
Fcα /μ R	In humans, FDCs are the predominant cell type expressing Fcα /μ R which are involved in IC retention (El Shikh et al., 2009b; Honda et al., 2009).
FDC-M1 (Mfge8)	Fat globule epidermal growth factor 8 (Mfge8) “licenses” tingible body macrophages (TBMs) to engulf apoptotic bodies in GCs and helps minimize autoimmunity (Kranich et al., 2008).
FDC-M2 (C4b eptiope)	C4b binding protein (C4bBP) binds C4b and co-localizes with ICs on FDCs. FDC-C4BP has been shown to signal B-cells via CD40, independent of T-cell CD40L (CD154). Injection of mice with FDC-M2 inhibits C4BP development and TI-GC development (Taylor et al., 2002).
Hedgehog (HH) ligand	Sonic Hedgehog (SHH) ligand is produced by FDCs within the GCs of lymphoid follicles, and SHH ligand protects GCer B-cells from apoptosis. GC B-cells express the HH receptors, and their survival is altered after inhibition of the HH signaling pathway (Sacedon et al., 2005; Ok et al., 2012).
IL-15	IL-15 is produced by FDCs and is captured by IL-15R on the surface of FDCs. Surface IL-15 is active and promotes GC-B-cell proliferation (Park et al., 2004), and human primary FDCs *in vitro*. In addition, blocking of FDC IL-15 signaling reduced FDC secretion of CCL-2, CCL-5, CXCL-5 and CXCL-8, suggesting potentially important roles for recruitment of other cellular components required for GC reaction (Gil et al., 2010).
IL-6	FDCs are the source of IL-6 in GCs. Engaging FDC-Fcγ RIIB by ICs activates FDCs and enhances FDC-IL-6 production. FDC-IL-6 promotes GC development, IgG production, and SHM and terminal B-cell differentiation (Wu et al., 2009).
IL-7	IL-7 has been found in isolated tonsilar FDCs using RT-PCR and cell staining. IL-7 signaling coupled with crosslinking of surface immunoglobulin receptors results in B-cell proliferation (Kroncke et al., 1996).
Notch ligands	Notch ligands, Delta-like1 and Jagged 1, are expressed by FDCs and support GC-B-cell growth and survival (Yoon et al., 2009).
Prostaglandins (PGs)	The role of PGs in regulation of the GC reaction has been suggested in the late 90s. TBM were found to be rich in PGs via which they can downregulate the GC reaction (Smith et al., 1998). Recently, FDCs are identified as a source of PGs. Human FDC-HK cell line produce PGE2 that is inhibted by COX-2 depletion (Cho et al., 2011a) and IL-4 via JAK1 and STAT6 signaling pathway (Cho et al., 2011b, 2012b). Human FDC-PGs significantly enhance the expression levels of CD54, CD80, and CD86 on the surface of activated B-cells and augment their Ag presentation activity (Kim et al., 2012b). Using a mouse FDC line, it was also shown that IL-21 induces PGE2 secretion by FDCs (Magari et al., 2011).
TNFα-R and LT-R	FDC development and maturation (Allen and Cyster, 2008)
TNF-α	FDCs produced soluble TNF-α that promotos GC T-cell activation (Thacker et al., 2009).
Toll-like receptors (TLRs)	Dramatic upregulation of FDC-ICAM-1, VCAM-1, and FcγRIIB is observed after injecting LPS into animals expressing WT TLR4 but not in animals with mutated TLR4. Incubation of FDCs with LPS in vitro upregulates FcγRIIB, ICAM-1, and VCAM-1. FDCs express mRNA for TLR2, 3, 4 and 9 and injection of poly I:C brings up FDC-Fcγ RIIB to levels comparable with LPS (El Shikh et al., 2007b). FDC activation via TLR4 enhances isotype switching, somatic hypermutation, and the production of high-affinity Ig (Deshane and Chaplin, 2010; Garin et al., 2010).
Wnt5a	FDCs secrete Wnt5a and its production is upregulated by polyI:C. FDC-Wnt5a is a GC B-cell survival factor and might be a potential target for the regulation of B-cell immunity (Kim et al., 2012a).

#### FDC activation

Expression of FDC surface and secreted molecules is subject to regulation, and engagement with ICs, TLR ligands, or collagen type 1 induces FDC activation and upregulation of these molecules. In the GCs, FDCs bear high levels of Fcγ RIIB, ICAM-1, and VCAM-1, and these molecules are involved in converting poorly immunogenic ICs into a highly immunogenic form and facilitate FDC-B-cell interactions. FDC-trapped ICs (El Shikh et al., 2006; Smith and Clatworthy, 2010), TLR ligands (El Shikh et al., 2007b; Garin et al., 2010), and collagen type 1 (El Shikh et al., 2007a) differentially upregulate the FDC accessory molecules ICAM-1, VCAM-1, FcγRIIB, C4bBP, BAFF, IL-1β, IL-10, and IL-6 (El Shikh et al., 2007b; Wu et al., 2009; Garin et al., 2010).

Activated FDCs provide Ags to B-cells in a highly immunogenic form by: (1) multimerizing the Ags thus extensively crosslink multiple BCRs (El Shikh et al., 2009a,b, 2010); (2) minimizing the inhibitory ITIM signaling in B-cells (Aydar et al., 2004b; El Shikh et al., 2006, 2010); and (3) providing B-cell homing, survival, and costimulation signals in efficient FDC-B-cell synapses (El Shikh et al., 2009b, 2010).

A major consequence of activation is a marked increase in accessory activity. Both specific Ab responses and SHM are dramatically enhanced when FDCs are activated by TLR ligands suggesting that adjuvant activity likely involves FDC-activation and not just DC activation for T-cell co-stimulation (El Shikh et al., 2007b; Wu et al., 2008). Moreover, TLR-mediated stimulation of Peyer's patches' FDCs by bacterial products in the gut induces FDC-CXCL13, -BAFF, and -TGF-β 1 secretion and enhances IgA production. Via TGF- β 1, IgA, and other tissue-protective, anti-inflammatory, and anti-tumor molecules, TLR-stimulated FDCs uniquely regulate mucosal homeostasis and prevent excessive activation that causes inflammation, autoimmunity, or tumor formation in the gut (Suzuki et al., 2010). Recently, the non-toxic CTA1-DD adjuvant hosting the ADP-ribosylating CTA1 subunit from cholera toxin and a dimer of the D fragment from *Staphylococcus aureus* protein A was targeted to the FDC networks and its deposition appeared to be complement-dependent. The adjuvant directly activates complement, enabling binding of the adjuvant to the FDC, which strongly promoted the GC reaction, leading to augmented serum Ab titers and long-term memory development. The mechanism of FDC activation by CTA1-DD may be TLR-dependent or independent and this has to be further verified (Mattsson et al., 2011).

## FDCs in the pathogenesis of diseases

In addition to their contributions in regulated immunity, FDCs also play important roles in pathological states, including HIV/AIDS, prion diseases, autoimmunity and B-cell lymphomas.

### FDCs in autoimmune diseases

Autoimmune disorders frequently display follicles with IC-bearing FDCs, autoreactive GCs, and ongoing affinity maturation (Aloisi and Pujol-Borrell, 2006; Manzo et al., 2010). Moreover, FDCs retain ICs for years, and provide constant Ag depot for memory B-cell re-stimulation (Klaus et al., 1980; Tew et al., 1980; MacLennan and Gray, 1986; Gray, 1993; McHeyzer-Williams et al., 1993; Bachmann and Jennings, 2010); and interference with FDC-reticula attenuates autoreactive GC formation, reduces pathogenic auto-Ab titers and memory B lymphocytes and ameliorates arthritis (Anolik et al., 2008; Victoratos and Kollias, 2009; Vinuesa et al., 2009; Manzo et al., 2010). It has been recently demonstrated that FDC follicular units in rheumatoid arthritis synovium invariably express AID and are surrounded by anti-citrullinated protein Ab (ACPA)-producing plasma cells. This was further confirmed by evidence of sustained AID expression, B-cell proliferation, ongoing CSR, and production of human IgG ACPA from GC synovial tissue transplanted into SCID mice, independently of new B-cell influx from the systemic circulation (Humby et al., 2009; Manzo et al., 2010).

Data in various models and human studies suggests the requirement for auto-Ag to sustain the autoimmune response and that auto-Ag withdrawal inhibits it (Bach et al., 1998). FDCs present Ags to B-cells in a highly immunogenic form and membrane-bound Ags seem to be the predominant form of Ag that mediates B-cell activation *in vivo* (Harwood and Batista, 2010).

In contrast to self-Ags of low valency such as small soluble proteins, multivalent Ags crosslink BCRs, maintain downstream signaling, and induce B-cell activation. In addition, tolerized B-cells exhibit enhanced rates of BCR-mediated Ag uptake. However, when the Ag is repetitive, stable, and resistant to BCR-mediated endocytosis, like multimerized FDC-bound Ags, BCRs aggregate and signaling is maintained. This results in clonal expansion and Ab production that is dependent on the multitude of BCR signalosomes activated by extensive crosslinking of BCRs (Hinton et al., 2008). The high levels of FcγRIIB on FDCs protects the immunogenicity of FDC-ICs by minimizing serious inhibition of B-cell activation upon BCR/FcγRIIB crosslinking (Aydar et al., 2003, 2004b; El Shikh et al., 2006, 2009a). In fact, the expression of FcγRIIB is significantly reduced on rheumatoid arthritis memory B-cells and plasmablasts and these alterations on FcγRIIB were associated with high levels of anti-citrullinated vimentin auto-Abs (Catalan et al., 2010).

Not only FDCs present Ags in a periodic multivalent ICs, but also they provide complement-mediated B-cell co-stimulation (Qin et al., 1998; Aydar et al., 2002; Barrington et al., 2002). CR2-mediated complementation of BCR signals can overcome B-cell anergy (Lyubchenko et al., 2007), and activation of the complement system is involved in the pathogenesis of the systemic autoimmune diseases via the classical pathway and the alternative pathways (Chen et al., 2010).

Previous *in vitro* studies indicated that peripheral blood B-cells from rheumatoid arthritis patients as well as healthy controls can be induced *in vitro* to produce anti-CCP Abs using CD40L polyclonal stimulation or in co-cultures with anti-CD3-activated T-cells (Reparon-Schuijt et al., 2001). FDC-C4bBP is a B-cell CD40L on activated FDCs, and infections at the onset of many autoimmune diseases may directly activate FDCs by ligating TLRs, and thus enhance FDC-B-cell interaction (El Shikh et al., 2007b).

The initial Ab needed to trap Ag on FDCs may be induced by molecular mimics or by polyclonal B-cell activation, which may occur as a consequence of infections that herald many autoimmune diseases (Granholm and Cavallo, 1991; Sfriso et al., 2010). However, once the ICs form, they would be trapped by FDCs and the autoreactive B-cells should be stimulated in a TI manner as we have shown for foreign Ags in the absence of T-cells (El Shikh et al., 2009a, 2010).

Polyvalent presentation of self-proteins in repetitive arrays breaches B-cell tolerance in immune-tolerant animal models (Sauerborn et al., 2010). Aggregated native hIFN-α broke B-cell tolerance in transgenic mice (Hermeling et al., 2006), and virus-like protein-arrayed HEL induced higher titer Ab responses against HEL in tolerant HEL Tg mice (Chackerian et al., 2008; Link and Bachmann, 2010). In certain occasions the high epitope valency rendered Ab responses independent of CD21 (Jegerlehner et al., 2002). Moreover, in humans, active immunization using virus-like particle-based vaccines selectively targeting IL-17A, soluble TNF-α or IL-1β has shown promising results in the protection against autoimmune arthritis and myocarditis in mouse models (Spohn et al., 2005; Rohn et al., 2006; Sonderegger et al., 2006; Spohn et al., 2007, 2008; Kopf et al., 2010).

### FDCs in HIV/AIDS

The contributions of FDCs to HIV pathogenesis include the storage of a large and diverse reservoir of infectious virus, including drug resistant variants, in an activated microenvironment where CD4 T-cells targets are attracted but impaired in their ability to leave thereby increasing the likelihood of their infection. Just as Ags, intact HIV particles are found trapped on FDC dendritic presented in the form of ICs containing Ab and/or complement proteins. FDCs retain virus particles for long periods of time and, in fact, as long as FDCs are present in infected individuals, HIV can be found associated with them (Heath et al., 1995; Masuda et al., 1995; Burton et al., 1997; Smith et al., 2001; Smith-Franklin et al., 2002).

FDCs provide a microenvironment that is highly conducive to HIV transmission. HIV ICs remain infectious for months, and HIV on FDCs is “presented” to T-cells resulting in propagation of infection. FDC-secreted TNF-α increases the rate of HIV transcription by about three-fold in infected T-cells resulting in a significant increase in virus production (Thacker et al., 2009). FDCs also increase the T-cell expression of the HIV coreceptor CXCR4 and these cells become highly susceptible to infection with small quantities of virus that do not infect other cells with lower levels of CXCR4. FDC-produced CXCL13 also contributes to HIV pathogenesis by attracting HIV-susceptible T lymphocytes into the lymphoid follicle (Estes et al., 2004).

### FDCs in prion diseases

FDCs appear to play an important role in prion diseases as evidenced by the finding that in experimental models where FDCs are not present, prion diseases do not typically appear. Prion diseases occur in both animals and humans and are caused by misfolded proteins encoded by the Prnp gene and consist of a normal form (PrPc) and its aberrant counterpart (PrPSc). Without the normal PrPc protein, whose function is still unknown, the diseases are not manifest (Nuvolone et al., 2009; Aguzzi and Krautler, 2010).

Upon exposure to prions, the agents are transported into secondary lymphoid tissues where the proteins accumulate and replicate on FDCs. FDCs is a major source of PrPc and complement proteins play critical role in its retention. PrPc-expressing FDCs are sufficient to sustain prion replication in the spleen (McCulloch et al., 2011).

After prion replication in the secondary lymphoid tissues, the PrPc-PRPSc complexes are transported to the central nervous system where neurodegeneration occurs. The exact mechanism of prion transport from FDCs to peripheral nerves is unknown. Prions could be (1) transported by various cell types leaving GCs toward nerve terminals; (2) incorporated by budding retroviruses; (3) released in FDC-derived iccosomes, or (4) passively diffuse from the site of replication to the site of peripheral innervation (Nuvolone et al., 2009). Recent studies indicate that B-cells interacting with and acquiring surface proteins from FDCs and recirculating between secondary lymphoid tissues via the blood and lymph are involved in the initial propagation of prions from the draining lymphoid tissue to peripheral tissues (Mok et al., 2012). Because FDCs appear to be required for disease pathogenesis in most cases, blocking agents to the LTβ R pathway, needed for FDC development and maintenance, results in impaired development of disease suggesting the potential of this approach in therapy (Nuvolone et al., 2009; Aguzzi and Krautler, 2010).

### FDCs in B-cell lymphomas

Follicular lymphomas morphologically resemble conventional GCs having B-cells, T-cells, macrophages, and FDCs. Recent studies suggest that the microenvironment in which the malignant cells reside is critical for disease pathogenesis and progression. The malignant cells appear to require the contributions of T-cells, macrophages, and FDCs as evidenced by the observation that in the absence of these cells, the malignant cells are difficult to survive. In addition to providing signals important to tumor cell growth, FDCs also can provide signals that spare malignant B-cells from undergoing apoptosis or programmed cell death. Moreover, FDCs establish a protective sanctuary for malignant cells from otherwise effective chemotherapeutic agents (Asadullah et al., 2000; Li et al., 2004; Kim et al., 2012a; Lee et al., 2012; Ok et al., 2012).

## Concluding comments

FDCs represent a unique accessory immune cell that provides both Ag-driven and co-signals to B-cells. FDC-derived signals contribute to the ability of B-cells to survive, proliferate, differentiate, and produce optimal levels of specific high affinity Abs during the GC reaction. Apart from their immunoregulatory functions, FDCs also contribute to several disease conditions including autoimmune diorders, AIDS, prion diseases, and follicular lymphomas. Further understanding of FDC biology is essential for better control of humoral immunity and paves the way for therapeutic management of FDC-mediated immune disorders.

### Conflict of interest statement

The authors declare that the research was conducted in the absence of any commercial or financial relationships that could be construed as a potential conflict of interest.

## References

[B1] Aguzzi A. Krautler N. J. (2010). Characterizing follicular dendritic cells: a progress report. Eur. J. Immunol. 40, 2134–2138 10.1002/eji.201040765 20853499

[B2] Allen C. D. Cyster J. G. (2008). Follicular dendritic cell networks of primary follicles and germinal centers: phenotype and function. Semin. Immunol. 20, 14–25 10.1016/j.smim.2007.12.001 18261920 PMC2366796

[B3] Allen C. D. Okada T. Cyster J. G. (2007). Germinal-center organization and cellular dynamics. Immunity 27, 190–202 10.1016/j.immuni.2007.07.009 17723214 PMC2242846

[B4] Aloisi F. Pujol-Borrell R. (2006). Lymphoid neogenesis in chronic inflammatory diseases. Nat. Rev. Immunol. 6, 205–217 10.1038/nri1786 16498451

[B5] Anolik J. H. Ravikumar R. Barnard J. Owen T. Almudevar A. Milner E. C. Miller C. H. Dutcher P. O. Hadley J. A. Sanz I. (2008). Cutting edge: anti-tumor necrosis factor therapy in rheumatoid arthritis inhibits memory B lymphocytes via effects on lymphoid germinal centers and follicular dendritic cell networks. J. Immunol. 180, 688–692 18178805 10.4049/jimmunol.180.2.688

[B6] Ansel K. M. Ngo V. N. Hyman P. L. Luther S. A. Forster R. Sedgwick J. D. Browning J. L. Lipp M. Cyster J. G. (2000). A chemokine-driven positive feedback loop organizes lymphoid follicles. Nature 406, 309–314 10.1038/35018581 10917533

[B7] Asadullah K. Gellrich S. Haeussler-Quade A. Friedrich M. Docke W. D. Jahn S. Sterry W. (2000). Cytokine expression in primary cutaneous germinal center cell lymphomas. Exp. Dermatol. 9, 71–76 10.1034/j.1600-0625.2000.009001071.x 10688378

[B8] Aydar Y. Balogh P. Tew J. G. Szakal A. K. (2002). Age-related depression of FDC accessory functions and CD21 ligand-mediated repair of co-stimulation. Eur. J. Immunol. 32, 2817–2826 10.1002/1521-4141(2002010)32:10<2817::AID-IMMU2817>3.0.CO;2-Z 12355434

[B9] Aydar Y. Balogh P. Tew J. G. Szakal A. K. (2003). Altered regulation of Fc gamma RII on aged follicular dendritic cells correlates with immunoreceptor tyrosine-based inhibition motif signaling in B cells and reduced germinal center formation. J. Immunol. 171, 5975–5987 14634109 10.4049/jimmunol.171.11.5975

[B10] Aydar Y. Balogh P. Tew J. G. Szakal A. K. (2004a). Follicular dendritic cells in aging, a “bottle-neck” in the humoral immune response. Ageing Res. Rev. 3, 15–29 10.1016/j.arr.2003.08.002 15163101

[B11] Aydar Y. Wu J. Song J. Szakal A. K. Tew J. G. (2004b). FcgammaRII expression on follicular dendritic cells and immunoreceptor tyrosine-based inhibition motif signaling in B cells. Eur. J. Immunol. 34, 98–107 10.1002/eji.200324147 14971035

[B12] Aydar Y. Sukumar S. Szakal A. K. Tew J. G. (2005). The influence of immune complex-bearing follicular dendritic cells on the IgM response, Ig class switching, and production of high affinity IgG. J. Immunol. 174, 5358–5366 15843533 10.4049/jimmunol.174.9.5358

[B13] Bach J. F. Koutouzov S. van Endert P. M. (1998). Are there unique autoantigens triggering autoimmune diseases? Immunol. Rev. 164, 139–155 9795772 10.1111/j.1600-065x.1998.tb01216.x

[B14] Bachmann M. F. Jennings G. T. (2010). Vaccine delivery: a matter of size, geometry, kinetics and molecular patterns. Nat. Rev. Immunol. 10, 787–796 10.1038/nri2868 20948547

[B15] Bachmann M. F. Zinkernagel R. M. (1997). Neutralizing antiviral B cell responses. Annu. Rev. Immunol. 15, 235–270 10.1146/annurev.immunol.15.1.235 9143688

[B16] Bajenoff M. Germain R. N. (2009). B-cell follicle development remodels the conduit system and allows soluble antigen delivery to follicular dendritic cells. Blood 114, 4989–4997 10.1182/blood-2009-06-229567 19713459 PMC2788973

[B17] Barrington R. A. Pozdnyakova O. Zafari M. R. Benjamin C. D. Carroll M. C. (2002). B lymphocyte memory: role of stromal cell complement and FcgammaRIIB receptors. J. Exp. Med. 196, 1189–1199 10.1084/jem.20021110 12417629 PMC2194107

[B18] Baumgarth N. (2000). A two-phase model of B-cell activation. Immunol. Rev. 176, 171–180 10.1034/j.1600-065X.2000.00606.x 11043776

[B19] Brunswick M. Burkhardt A. Finkelman F. Bolen J. Mond J. J. (1992). Comparison of tyrosine kinase activation by mitogenic and nonmitogenic anti-IgD antibodies. J. Immunol. 149, 2249–2254 1326578

[B20] Brunswick M. Finkelman F. D. Highet P. F. Inman J. K. Dintzis H. M. Mond J. J. (1988). Picogram quantities of anti-Ig antibodies coupled to dextran induce B cell proliferation. J. Immunol. 140, 3364–3372 2452184

[B21] Burton G. F. Conrad D. H. Szakal A. K. Tew J. G. (1993). Follicular dendritic cells and B cell costimulation. J. Immunol. 150, 31–38 8417129

[B22] Burton G. F. Masuda A. Heath S. L. Smith B. A. Tew J. G. Szakal A. K. (1997). Follicular dendritic cells (FDC) in retroviral infection: host/pathogen perspectives. Immunol. Rev. 156, 185–197 9176708 10.1111/j.1600-065x.1997.tb00968.x

[B23] Carragher D. M. Rangel-Moreno J. Randall T. D. (2008). Ectopic lymphoid tissues and local immunity. Semin. Immunol. 20, 26–42 10.1016/j.smim.2007.12.004 18243731 PMC2276727

[B24] Carroll M. C. (1998). The role of complement and complement receptors in induction and regulation of immunity. Annu. Rev. Immunol. 16, 545–568 10.1146/annurev.immunol.16.1.545 9597141

[B25] Carter R. H. Doody G. M. Bolen J. B. Fearon D. T. (1997). Membrane IgM-induced tyrosine phosphorylation of CD19 requires a CD19 domain that mediates association with components of the B cell antigen receptor complex. J. Immunol. 158, 3062–3069 9120258

[B26] Catalan D. Aravena O. Sabugo F. Wurmann P. Soto L. Kalergis A. M. Cuchacovich M. Aguillon J. C. (2010). B cells from rheumatoid arthritis patients show important alterations in the expression of CD86 and FcgammaRIIb, which are modulated by anti-tumor necrosis factor therapy. Arthritis Res. Ther. 12, R68 10.1186/ar2985 20398308 PMC2888223

[B27] Chackerian B. Durfee M. R. Schiller J. T. (2008). Virus-like display of a neo-self antigen reverses B cell anergy in a B cell receptor transgenic mouse model. J. Immunol. 180, 5816–5825 18424700 10.4049/jimmunol.180.9.5816 PMC3493123

[B28] Chaimowitz N. S. Martin R. K. Cichy J. Gibb D. R. Patil P. Kang D. J. Farnsworth J. Butcher E. C. McCright B. Conrad D. H. (2011). A disintegrin and metalloproteinase 10 regulates antibody production and maintenance of lymphoid architecture. J. Immunol. 187, 5114–5122 10.4049/jimmunol.1102172 21998451 PMC4006936

[B29] Chan T. D. Brink R. (2012). Affinity-based selection and the germinal center response. Immunol. Rev. 247, 11–23 10.1111/j.1600-065X.2012.01118.x 22500828

[B30] Chen M. Daha M. R. Kallenberg C. G. (2010). The complement system in systemic autoimmune disease. J. Autoimmun. 34, J276–J286 10.1016/j.jaut.2009.11.014 20005073

[B31] Cho K. A. Kim J. Y. Kim H. S. Ryu K. H. Woo S. Y. (2012a). Tonsil-derived mesenchymal progenitor cells acquire a follicular dendritic cell phenotype under cytokine stimulation. Cytokine 59, 211–214 10.1016/j.cyto.2012.04.016 22578801

[B32] Cho W. Jeoung D. I. Kim Y. M. Choe J. (2012b). STAT6 and JAK1 are essential for IL-4-mediated suppression of prostaglandin production in human follicular dendritic cells: opposing roles of phosphorylated and unphosphorylated STAT6. Int. Immunopharmacol. 12, 635–642 10.1016/j.intimp.2012.02.012 22406175

[B33] Cho W. Choi J. Park C. H. Yoon S. O. Jeoung D. I. Kim Y. M. Choe J. (2008). Expression of CD320 in human B cells in addition to follicular dendritic cells. BMB Rep. 41, 863–867 19123977 10.5483/bmbrep.2008.41.12.863

[B34] Cho W. Kim J. Cho K. B. Choe J. (2011a). Production of prostaglandin e(2) and i(2) is coupled with cyclooxygenase-2 in human follicular dendritic cells. Immune. Netw. 11, 364–367 10.4110/in.2011.11.6.364 22346776 PMC3275705

[B35] Cho W. Kim Y. Jeoung D. I. Kim Y. M. Choe J. (2011b). IL-4 and IL-13 suppress prostaglandins production in human follicular dendritic cells by repressing COX-2 and mPGES-1 expression through JAK1 and STAT6. Mol. Immunol. 48, 966–972 10.1016/j.molimm.2011.01.007 21277633

[B36] Cinamon G. Zachariah M. A. Lam O. M. Foss F. W. Jr. Cyster J. G. (2008). Follicular shuttling of marginal zone B cells facilitates antigen transport. Nat. Immunol. 9, 54–62 10.1038/ni1542 18037889 PMC2488964

[B37] Cozine C. L. Wolniak K. L. Waldschmidt T. J. (2005). The primary germinal center response in mice. Curr. Opin. Immunol. 17, 298–302 10.1016/j.coi.2005.04.007 15886120

[B38] Croix D. A. Ahearn J. M. Rosengard A. M. Han S. Kelsoe G. Ma M. Carroll M. C. (1996). Antibody response to a T-dependent antigen requires B cell expression of complement receptors. J. Exp. Med. 183, 1857–1864 8666942 10.1084/jem.183.4.1857 PMC2192488

[B39] Crotty S. (2011). Follicular helper CD4 T cells (TFH). Annu. Rev. Immunol. 29, 621–663 10.1146/annurev-immunol-031210-101400 21314428

[B40] Cyster J. G. (2010). B cell follicles and antigen encounters of the third kind. Nat. Immunol. 11, 989–996 10.1038/ni.1946 20959804

[B41] Cyster J. G. Ansel K. M. Reif K. Ekland E. H. Hyman P. L. Tang H. L. Luther S. A. Ngo V. N. (2000). Follicular stromal cells and lymphocyte homing to follicles. Immunol. Rev. 176, 181–193 10.1034/j.1600-065X.2000.00618.x 11043777

[B42] Deng C. Goluszko E. Tuzun E. Yang H. Christadoss P. (2002). Resistance to experimental autoimmune myasthenia gravis in IL-6-deficient mice is associated with reduced germinal center formation and C3 production. J. Immunol. 169, 1077–1083 12097416 10.4049/jimmunol.169.2.1077

[B43] Deshane J. Chaplin D. D. (2010). Follicular dendritic cell makes environmental sense. Immunity 33, 2–4 10.1016/j.immuni.2010.07.008 20643332 PMC2919488

[B44] de Vinuesa C. G. Cook M. C. Ball J. Drew M. Sunners Y. Cascalho M. Wabl M. Klaus G. G. MacLennan I. C. (2000). Germinal centers without T cells. J. Exp. Med. 191, 485–494 10662794 10.1084/jem.191.3.485 PMC2195827

[B45] Dintzis H. M. Dintzis R. Z. Vogelstein B. (1976). Molecular determinants of immunogenicity: the immunon model of immune response. Proc. Natl. Acad. Sci. U.S.A. 73, 3671–3675 62364 10.1073/pnas.73.10.3671 PMC431180

[B46] Dintzis R. Z. Middleton M. H. Dintzis H. M. (1983). Studies on the immunogenicity and tolerogenicity of T-independent antigens. J. Immunol. 131, 2196–2203 6631009

[B47] Dintzis R. Z. Vogelstein B. Dintzis H. M. (1982). Specific cellular stimulation in the primary immune response: experimental test of a quantized model. Proc. Natl. Acad. Sci. U.S.A. 79, 884–888 6950432 10.1073/pnas.79.3.884 PMC345857

[B48] Donaldson S. L. Kosco M. H. Szakal A. K. Tew J. G. (1986). Localization of antibody-forming cells in draining lymphoid organs during long-term maintenance of the antibody response. J. Leukoc. Biol. 40, 147–157 3461094 10.1002/jlb.40.2.147

[B49] Elgueta R. de Vries V. C. Noelle R. J. (2010). The immortality of humoral immunity. Immunol. Rev. 236, 139–150 10.1111/j.1600-065X.2010.00924.x 20636814

[B50] El Shikh M. E. El Sayed R. Szakal A. K. Tew J. G. (2006). Follicular dendritic cell (FDC)-FcgammaRIIB engagement via immune complexes induces the activated FDC phenotype associated with secondary follicle development. Eur. J. Immunol. 36, 2715–2724 10.1002/eji.200636122 17013985

[B51] El Shikh M. E. El Sayed R. M. Sukumar S. Szakal A. K. Tew J. G. (2010). Activation of B cells by antigens on follicular dendritic cells. Trends Immunol. 31, 205–211 10.1016/j.it.2010.03.002 20418164 PMC2886728

[B52] El Shikh M. E. El Sayed R. M. Szakal A. K. Tew J. G. (2009a). T-independent antibody responses to T-dependent antigens: a novel follicular dendritic cell-dependent activity. J. Immunol. 182, 3482–3491 10.4049/jimmunol.0802317 19265126

[B53] El Shikh M. E. El Sayed R. M. Tew J. G. Burton G. F. (2009b). Follicular Dendritic Cells (B Lymphocyte Stimulating). Encyclopedia of Life Sciences. Chichester: John Wiley and Sons, Ltd

[B54] El Shikh M. E. El Sayed R. M. Tew J. G. Szakal A. K. (2007a). Follicular dendritic cells stimulated by collagen type I develop dendrites and networks *in vitro*. Cell Tissue Res. 329, 81–89 10.1007/s00441-007-0394-6 17372768

[B55] El Shikh M. E. El Sayed R. M. Wu Y. Szakal A. K. Tew J. G. (2007b). TLR4 on follicular dendritic cells: an activation pathway that promotes accessory activity. J. Immunol. 179, 4444–4450 17878340 10.4049/jimmunol.179.7.4444

[B56] Estes J. D. Thacker T. C. Hampton D. L. Kell S. A. Keele B. F. Palenske E. A. Druey K. M. Burton G. F. (2004). Follicular dendritic cell regulation of CXCR4-mediated germinal center CD4 T cell migration. J. Immunol. 173, 6169–6178 15528354 10.4049/jimmunol.173.10.6169

[B57] Fakher M. Wu J. Qin D. Szakal A. Tew J. (2001). Follicular dendritic cell accessory activity crosses MHC and species barriers. Eur. J. Immunol. 31, 176–185 10.1002/1521-4141(200101)31:1<#60;176::AID-IMMU176>#62;3.0.CO;2-H 11169451

[B58] Fazilleau N. Mark L. McHeyzer-Williams L. J. McHeyzer-Williams M. G. (2009). Follicular helper T cells: lineage and location. Immunity 30, 324–335 10.1016/j.immuni.2009.03.003 19303387 PMC2731675

[B59] Fehr T. Bachmann M. F. Bucher E. Kalinke U. Di Padova F. E. Lang A. B. Hengartner H. Zinkernagel R. M. (1997). Role of repetitive antigen patterns for induction of antibodies against antibodies. J. Exp. Med. 185, 1785–1792 10.1084/jem.185.10.1785 9151704 PMC2196322

[B60] Ferguson A. R. Youd M. E. Corley R. B. (2004). Marginal zone B cells transport and deposit IgM-containing immune complexes onto follicular dendritic cells. Int. Immunol. 16, 1411–1422 10.1093/intimm/dxh142 15326094

[B61] Fischer M. B. Ma M. Goerg S. Zhou X. Xia J. Finco O. Han S. Kelsoe G. Howard R. G. Rothstein T. L. Kremmer E. Rosen F. S. Carroll M. C. (1996). Regulation of the B cell response to T-dependent antigens by classical pathway complement. J. Immunol. 157, 549–556 8752901

[B62] Garin A. Meyer-Hermann M. Contie M. Figge M. T. Buatois V. Gunzer M. Toellner K. M. Elson G. Kosco-Vilbois M. H. (2010). Toll-like receptor 4 signaling by follicular dendritic cells is pivotal for germinal center onset and affinity maturation. Immunity 33, 84–95 10.1016/j.immuni.2010.07.005 20643339

[B63] Gaspal F. M. McConnell F. M. Kim M. Y. Gray D. Kosco-Vilbois M. H. Raykundalia C. R. Botto M. Lane P. J. (2006). The generation of thymus-independent germinal centers depends on CD40 but not on CD154, the T cell-derived CD40-ligand. Eur. J. Immunol. 36, 1665–1673 10.1002/eji.200535339 16783845

[B64] Gibb D. R. El Shikh M. Kang D. J. Rowe W. J. El Sayed R. Cichy J. Yagita H. Tew J. G. Dempsey P. J. Crawford H. C. Conrad D. H. (2010). ADAM10 is essential for Notch2-dependent marginal zone B cell development and CD23 cleavage *in vivo*. J. Exp. Med. 207, 623–635 10.1084/jem.20091990 20156974 PMC2839139

[B65] Gil M. Park S. J. Chung Y. S. Park C. S. (2010). Interleukin-15 enhances proliferation and chemokine secretion of human follicular dendritic cells. Immunology 130, 536–544 10.1111/j.1365-2567.2010.03252.x 20331472 PMC2913264

[B66] Gonzalez S. F. Degn S. E. Pitcher L. A. Woodruff M. Heesters B. A. Carroll M. C. (2011). Trafficking of B cell antigen in lymph nodes. Annu. Rev. Immunol. 29, 215–233 10.1146/annurev-immunol-031210-101255 21219172

[B67] Good-Jacobson K. L. Shlomchik M. J. (2010). Plasticity and heterogeneity in the generation of memory B cells and long-lived plasma cells: the influence of germinal center interactions and dynamics. J. Immunol. 185, 3117–3125 10.4049/jimmunol.1001155 20814029

[B68] Granholm N. A. Cavallo T. (1991). Bacterial lipopolysaccharide enhances deposition of immune complexes and exacerbates nephritis in BXSB lupus-prone mice. Clin. Exp. Immunol. 85, 270–277 1864008 10.1111/j.1365-2249.1991.tb05717.x PMC1535747

[B69] Gray D. (1993). Immunological memory: a function of antigen persistence. Trends Microbiol. 1, 39–41 8044459 10.1016/0966-842x(93)90026-n

[B70] Gray D. Skarvall H. (1988). B-cell memory is short-lived in the absence of antigen. Nature 336, 70–73 10.1038/336070a0 3263573

[B71] Groom J. R. Fletcher C. A. Walters S. N. Grey S. T. Watt S. V. Sweet M. J. Smyth M. J. Mackay C. R. Mackay F. (2007). BAFF and MyD88 signals promote a lupuslike disease independent of T cells. J. Exp. Med. 204, 1959–1971 10.1084/jem.20062567 17664289 PMC2118661

[B72] Guzman-Rojas L. Sims-Mourtada J. C. Rangel R. Martinez-Valdez H. (2002). Life and death within germinal centres: a double-edged sword. Immunology 107, 167–175 10.1046/j.1365-2567.2002.01494.x 12383195 PMC1782796

[B73] Harwood N. E. Batista F. D. (2010). Early events in B cell activation. Annu. Rev. Immunol. 28, 185–210 10.1146/annurev-immunol-030409-101216 20192804

[B74] Hase H. Kanno Y. Kojima M. Hasegawa K. Sakurai D. Kojima H. Tsuchiya N. Tokunaga K. Masawa N. Azuma M. Okumura K. Kobata T. (2004). BAFF/BLyS can potentiate B-cell selection with the B-cell coreceptor complex. Blood 103, 2257–2265 10.1182/blood-2003-08-2694 14630796

[B75] Heath S. L. Tew J. G. Tew J. G. Szakal A. K. Burton G. F. (1995). Follicular dendritic cells and human immunodeficiency virus infectivity. Nature 377, 740–744 10.1038/377740a0 7477265

[B76] Heijink I. H. Vellenga E. Borger P. Postma D. S. de Monchy J. G. Kauffman H. F. (2002). Interleukin-6 promotes the production of interleukin-4 and interleukin-5 by interleukin-2-dependent and -independent mechanisms in freshly isolated human T cells. Immunology 107, 316–324 10.1046/j.1365-2567.2002.01501.x 12423307 PMC1782800

[B77] Hermeling S. Schellekens H. Maas C. Gebbink M. F. Crommelin D. J. Jiskoot W. (2006). Antibody response to aggregated human interferon alpha2b in wild-type and transgenic immune tolerant mice depends on type and level of aggregation. J. Pharm. Sci. 95, 1084–1096 10.1002/jps.20599 16552750

[B78] Hinton H. J. Jegerlehner A. Bachmann M. F. (2008). Pattern recognition by B cells: the role of antigen repetitiveness versus Toll-like receptors. Curr. Top. Microbiol. Immunol. 319, 1–15 18080412 10.1007/978-3-540-73900-5_1

[B79] Hollowood K. Goodlad J. R. (1998). Germinal centre cell kinetics. J. Pathol. 185, 229–233 10.1002/(SICI)1096-9896(199807)185:3<229::AID-PATH86>3.0.CO;2-L 9771474

[B80] Honda S. Kurita N. Miyamoto A. Cho Y. Usui K. Takeshita K. Takahashi S. Yasui T. Kikutani H. Kinoshita T. Fujita T. Tahara-Hanaoka S. Shibuya K. Shibuya A. (2009). Enhanced humoral immune responses against T-independent antigens in Fc alpha/muR-deficient mice. Proc. Natl. Acad. Sci. U.S.A. 106, 11230–11235 10.1073/pnas.0809917106 19549827 PMC2699373

[B81] Huard B. Arlettaz L. Ambrose C. Kindler V. Mauri D. Roosnek E. Tschopp J. Schneider P. French L. E. (2004). BAFF production by antigen-presenting cells provides T cell co-stimulation. Int. Immunol. 16, 467–475 10.1093/intimm/dxh043 14978020

[B82] Huard B. Schneider P. Mauri D. Tschopp J. French L. E. (2001). T cell costimulation by the TNF ligand BAFF. J. Immunol. 167, 6225–6231 11714784 10.4049/jimmunol.167.11.6225

[B83] Humby F. Bombardieri M. Manzo A. Kelly S. Blades M. C. Kirkham B. Spencer J. Pitzalis C. (2009). Ectopic lymphoid structures support ongoing production of class-switched autoantibodies in rheumatoid synovium. PLoS Med. 6:e1 10.1371/journal.pmed.0060001 19143467 PMC2621263

[B84] Jegerlehner A. Storni T. Lipowsky G. Schmid M. Pumpens P. Bachmann M. F. (2002). Regulation of IgG antibody responses by epitope density and CD21-mediated costimulation. Eur. J. Immunol. 32, 3305–3314 10.1002/1521-4141(200211)32:11<3305::AID-IMMU3305>3.0.CO;2-J 12555676

[B85] Jeurissen A. Ceuppens J. L. Bossuyt X. (2004). T lymphocyte dependence of the antibody response to ‘T lymphocyte independent type 2’ antigens. Immunology 111, 1–7 10.1111/j.1365-2567.2004.01775.x 14678191 PMC1782396

[B86] Kallies A. Hasbold J. Tarlinton D. M. Dietrich W. Corcoran L. M. Hodgkin P. D. Nutt S. L. (2004). Plasma cell ontogeny defined by quantitative changes in blimp-1 expression. J. Exp. Med. 200, 967–977 10.1084/jem.20040973 15492122 PMC2211847

[B87] Kapasi Z. F. Burton G. F. Shultz L. D. Tew J. G. Szakal A. K. (1993a). Cellular requirements for functional reconstitution of follicular dendritic cells in SCID mice. Adv. Exp. Med. Biol. 329, 383–386 8379399 10.1007/978-1-4615-2930-9_64

[B88] Kapasi Z. F. Burton G. F. Shultz L. D. Tew J. G. Szakal A. K. (1993b). Induction of functional follicular dendritic cell development in severe combined immunodeficiency mice. Influence of B and T cells. J. Immunol. 150, 2648–2658 8454847

[B89] Kapasi Z. F. Kosco-Vilbois M. H. Shultz L. D. Tew J. G. Szakal A. K. (1994). Cellular origin of follicular dendritic cells. Adv. Exp. Med. Biol. 355, 231–235 7709828 10.1007/978-1-4615-2492-2_39

[B90] Kapasi Z. F. Qin D. Kerr W. G. Kosco-Vilbois M. H. Shultz L. D. Tew J. G. Szakal A. K. (1998). Follicular dendritic cell (FDC) precursors in primary lymphoid tissues. J. Immunol. 160, 1078–1084 9570519

[B91] Kasajima-Akatsuka N. Maeda K. (2006). Development, maturation and subsequent activation of follicular dendritic cells (FDC): immunohistochemical observation of human fetal and adult lymph nodes. Histochem. Cell Biol. 126, 261–273 10.1007/s00418-006-0157-6 16470387

[B92] Kim J. Kim D. W. Chang W. Choe J. Kim J. Park C. S. Song K. Lee I. (2012a). Wnt5a is secreted by follicular dendritic cells to protect germinal center B cells via Wnt/Ca2+/NFAT/NF-kappaB-B cell lymphoma 6 signaling. J. Immunol. 188, 182–189 10.4049/jimmunol.1102297 22124122

[B93] Kim J. Kim Y. M. Jeoung D. I. Choe J. (2012b). Human follicular dendritic cells promote the APC capability of B cells by enhancing CD86 expression levels. Cell. Immunol. 273, 109–114 10.1016/j.cellimm.2012.01.003 22321156

[B94] Klaus G. G. Humphrey J. H. (1977). The generation of memory cells. I. The role of C3 in the generation of B memory cells. Immunology 33, 31–40 301502 PMC1445413

[B95] Klaus G. G. Humphrey J. H. Kunkl A. Dongworth D. W. (1980). The follicular dendritic cell: its role in antigen presentation in the generation of immunological memory. Immunol. Rev. 53, 3–28 7009406 10.1111/j.1600-065x.1980.tb01038.x

[B96] Kopf M. Bachmann M. F. Marsland B. J. (2010). Averting inflammation by targeting the cytokine environment. Nat. Rev. Drug Discov. 9, 703–718 10.1038/nrd2805 20811382

[B97] Kopf M. Herren S. Wiles M. V. Pepys M. B. Kosco-Vilbois M. H. (1998). Interleukin 6 influences germinal center development and antibody production via a contribution of C3 complement component. J. Exp. Med. 188, 1895–1906 10.1084/jem.188.10.1895 9815267 PMC2212418

[B98] Kosco M. H. Burton G. F. Kapasi Z. F. Szakal A. K. Tew J. G. (1989). Antibody-forming cell induction during an early phase of germinal centre development and its delay with ageing. Immunology 68, 312–318 2592007 PMC1385441

[B99] Kosco M. H. Pflugfelder E. Gray D. (1992). Follicular dendritic cell-dependent adhesion and proliferation of B cells in vitro. J. Immunol. 148, 2331–2339 1560196

[B100] Kosco-Vilbois M. H. (2003). Are follicular dendritic cells really good for nothing? Nat. Rev. Immunol. 3, 764–769 10.1038/nri1179 12949500

[B101] Kosco-Vilbois M. H. Bonnefoy J. Y. Chvatchko Y. (1997). The physiology of murine germinal center reactions. Immunol. Rev. 156, 127–136 9176704 10.1111/j.1600-065x.1997.tb00964.x

[B102] Kranich J. Krautler N. J. Heinen E. Polymenidou M. Bridel C. Schildknecht A. Huber C. Kosco-Vilbois M. H. Zinkernagel R. Miele G. Aguzzi A. (2008). Follicular dendritic cells control engulfment of apoptotic bodies by secreting Mfge8. J. Exp. Med. 205, 1293–1302 10.1084/jem.20071019 18490487 PMC2413028

[B103] Kroncke R. Loppnow H. Flad H. D. Gerdes J. (1996). Human follicular dendritic cells and vascular cells produce interleukin-7, a potential role for interleukin-7 in the germinal center reaction. Eur. J. Immunol. 26, 2541–2544 10.1002/eji.1830261040 8898972

[B104] Landy M. Baker P. J. (1966). Cytodynamics of the distinctive immune response produced in regional lymph nodes by Salmonella somatic polysaccharide. J. Immunol. 97, 670–679 5926455

[B105] Lee C. G. Das B. Lin T. L. Grimes C. Zhang X. Lavezzi T. Huang L. Cole J. Yau L. Li L. (2012). A rare fraction of drug-resistant follicular lymphoma cancer stem cells interacts with follicular dendritic cells to maintain tumourigenic potential. Br. J. Haematol. 158, 79–90 10.1111/j.1365-2141.2012.09123.x 22509798 PMC3374069

[B106] Lentz V. M. Manser T. (2001). Cutting edge: germinal centers can be induced in the absence of T cells. J. Immunol. 167, 15–20 11418626 10.4049/jimmunol.167.1.15

[B107] Leyva-Cobian F. Outschoorn I. M. Carrasco-Marin E. Alvarez-Dominguez C. (1997). The consequences of the intracellular retention of pathogen-derived T-cell-independent antigens on protein presentation to T cells. Clin. Immunol. Immunopathol. 85, 1–15 10.1006/clin.1997.4426 9325063

[B108] Li L. Yoon S. O. Fu D. D. Zhang X. Choi Y. S. (2004). Novel follicular dendritic cell molecule, 8D6, collaborates with CD44 in supporting lymphomagenesis by a Burkitt lymphoma cell line, L3055. Blood 104, 815–821 10.1182/blood-2004-01-0292 15090445

[B109] Link A. Bachmann M. F. (2010). Immunodrugs: breaking B- but not T-cell tolerance with therapeutic anticytokine vaccines. Immunotherapy 2, 561–574 10.2217/imt.10.30 20636009

[B110] Link A. Zabel F. Schnetzler Y. Titz A. Brombacher F. Bachmann M. F. (2012). Innate immunity mediates follicular transport of particulate but not soluble protein antigen. J. Immunol. 188, 3724–3733 10.4049/jimmunol.1103312 22427639

[B111] Linterman M. A. Liston A. Vinuesa C. G. (2012). T-follicular helper cell differentiation and the co-option of this pathway by non-helper cells. Immunol. Rev. 247, 143–159 10.1111/j.1600-065X.2012.01121.x 22500838

[B112] Lopes-Carvalho T. Foote J. Kearney J. F. (2005). Marginal zone B cells in lymphocyte activation and regulation. Curr. Opin. Immunol. 17, 244–250 10.1016/j.coi.2005.04.009 15886113

[B113] Lyubchenko T. Dal Porto J. M. Holers V. M. Cambier J. C. (2007). Cutting edge: complement (C3d)-linked antigens break B cell anergy. J. Immunol. 179, 2695–2699 17709481 10.4049/jimmunol.179.5.2695

[B114] Mabbott N. A. Kenneth B. J. Kobayashi A. Donaldson D. S. Ohmori H. Yoon S. O. Freedman A. S. Freeman T. C. Summers K. M. (2011). Expression of mesenchyme-specific gene signatures by follicular dendritic cells: insights from the meta-analysis of microarray data from multiple mouse cell populations. Immunology 133, 482–498 10.1111/j.1365-2567.2011.03461.x 21635249 PMC3143359

[B115] MacLennan I. C. (1994). Germinal centers. Annu. Rev. Immunol. 12, 117–139 10.1146/annurev.iy.12.040194.001001 8011279

[B116] MacLennan I. C. (2008). B cells: the follicular dimension of the marginal zone. Immunol. Cell Biol. 86, 219–220 10.1038/icb.2008.2 18301384

[B117] MacLennan I. C. Garcia d V. Casamayor-Palleja M. (2000). B-cell memory and the persistence of antibody responses. Philos. Trans. R. Soc. Lond. B Biol. Sci. 355, 345–350 10.1098/rstb.2000.0571 10794052 PMC1692743

[B118] MacLennan I. C. Gray D. (1986). Antigen-driven selection of virgin and memory B cells. Immunol. Rev. 91, 61–85 3089914 10.1111/j.1600-065x.1986.tb01484.x

[B119] Maeda K. Kosco-Vilbois M. H. Burton G. F. Szakal A. K. Tew J. G. (1995). Expression of the intercellular adhesion molecule-1 on high endothelial venules and on non-lymphoid antigen handling cells: interdigitating cells, antigen transporting cells and follicular dendritic cells. Cell Tissue Res. 279, 47–54 7895264 10.1007/BF00300690

[B120] Magari M. Nishikawa Y. Fujii Y. Nishio Y. Watanabe K. Fujiwara M. Kanayama N. Ohmori H. (2011). IL-21-dependent B cell death driven by prostaglandin E2, a product secreted from follicular dendritic cells. J. Immunol. 187, 4210–4218 10.4049/jimmunol.1100934 21911600

[B121] Magliozzi R. Columba-Cabezas S. Serafini B. Aloisi F. (2004). Intracerebral expression of CXCL13 and BAFF is accompanied by formation of lymphoid follicle-like structures in the meninges of mice with relapsing experimental autoimmune encephalomyelitis. J. Neuroimmunol. 148, 11–23 10.1016/j.jneuroim.2003.10.056 14975582

[B122] Mandel T. E. Phipps R. P. Abbot A. Tew J. G. (1980). The follicular dendritic cell: long term antigen retention during immunity. Immunol. Rev. 53, 29–59 6162778 10.1111/j.1600-065x.1980.tb01039.x

[B123] Manser T. (2004). Textbook germinal centers? J. Immunol. 172, 3369–3375 15004133 10.4049/jimmunol.172.6.3369

[B124] Manzo A. Bombardieri M. Humby F. Pitzalis C. (2010). Secondary and ectopic lymphoid tissue responses in rheumatoid arthritis: from inflammation to autoimmunity and tissue damage/remodeling. Immunol. Rev. 233, 267–285 10.1111/j.0105-2896.2009.00861.x 20193005

[B125] Manzo A. Paoletti S. Carulli M. Blades M. C. Barone F. Yanni G. Fitzgerald O. Bresnihan B. Caporali R. Montecucco C. Uguccioni M. Pitzalis C. (2005). Systematic microanatomical analysis of CXCL13 and CCL21 in situ production and progressive lymphoid organization in rheumatoid synovitis. Eur. J. Immunol. 35, 1347–1359 10.1002/eji.200425830 15832291

[B126] Masuda A. Burton G. F. Szakal A. K. Tew J. G. (1995). Loss of follicular dendritic cells in murine-acquired immunodeficiency syndrome. Lab. Invest. 73, 511–520 7474923

[B127] Mattsson J. Yrlid U. Stensson A. Schon K. Karlsson M. C. Ravetch J. V. Lycke N. Y. (2011). Complement activation and complement receptors on follicular dendritic cells are critical for the function of a targeted adjuvant. J. Immunol. 187, 3641–3652 10.4049/jimmunol.1101107 21880985

[B128] McCulloch L. Brown K. L. Bradford B. M. Hopkins J. Bailey M. Rajewsky K. Manson J. C. Mabbott N. A. (2011). Follicular dendritic cell-specific prion protein (PrP) expression alone is sufficient to sustain prion infection in the spleen. PLoS Pathog. 7:e1002402 10.1371/journal.ppat.1002402 22144895 PMC3228802

[B129] McHeyzer-Williams L. J. McHeyzer-Williams M. G. (2005). Antigen-specific memory B cell development. Annu. Rev. Immunol. 23, 487–513 10.1146/annurev.immunol.23.021704.115732 15771579

[B130] McHeyzer-Williams M. McHeyzer-Williams L. Panus J. Pogue-Caley R. Bikah G. Driver D. Eisenbraun M. (2003). Helper T-cell-regulated B-cell immunity. Microbes Infect. 5, 205–212 10.1016/S1286-4579(03)00012-1 12681409

[B131] McHeyzer-Williams M. Okitsu S. Wang N. McHeyzer-Williams L. (2012). Molecular programming of B cell memory. Nat. Rev. Immunol. 12, 24–34 10.1038/nri3128 22158414 PMC3947622

[B132] McHeyzer-Williams M. G. (2003). B cells as effectors. Curr. Opin. Immunol. 15, 354–361 10.1016/S0952-7915(03)00046-3 12787764

[B133] McHeyzer-Williams M. G. McHeyzer-Williams L. J. Fanelli P. J. Bikah G. Pogue-Caley R. R. Driver D. J. Eisenbraun M. D. (2000). Antigen-specific immunity. Th cell-dependent B cell responses. Immunol. Res. 22, 223–236 10.1385/IR:22:2-3:223 11339358

[B134] McHeyzer-Williams M. G. McLean M. J. Lalor P. A. Nossal G. J. (1993). Antigen-driven B cell differentiation *in vivo*. J. Exp. Med. 178, 295–307 8315385 10.1084/jem.178.1.295 PMC2191088

[B135] Moisini I. Davidson A. (2009). BAFF: a local and systemic target in autoimmune diseases. Clin. Exp. Immunol. 158, 155–163 10.1111/j.1365-2249.2009.04007.x 19737141 PMC2768805

[B136] Mok S. W. Proia R. L. Brinkmann V. Mabbott N. A. (2012). B cell-specific S1PR1 deficiency blocks prion dissemination between secondary lymphoid organs. J. Immunol. 188, 5032–5040 10.4049/jimmunol.1200349 22504650 PMC3364719

[B137] Moller G. (1965). 19S antibody production against soluble lipopolysaccharide antigens by individual lymphoid cells *in vitro*. Nature 207, 1166–1168 5326987 10.1038/2071166a0

[B138] Mond J. J. Lees A. Snapper C. M. (1995). T cell-independent antigens type 2. Annu. Rev. Immunol. 13, 655–692 10.1146/annurev.iy.13.040195.003255 7612238

[B139] Montes C. L. Acosta-Rodriguez E. V. Merino M. C. Bermejo D. A. Gruppi A. (2007). Polyclonal B cell activation in infections: infectious agents' devilry or defense mechanism of the host? J. Leukoc. Biol. 82, 1027–1032 10.1189/jlb.0407214 17615380

[B140] Mori A. Suko M. Kaminuma O. Inoue S. Ohmura T. Nishizaki Y. Nagahori T. Asakura Y. Hoshino A. Okumura Y. Sato G. Ito K. Okudaira H. (1996). IL-15 promotes cytokine production of human T helper cells. J. Immunol. 156, 2400–2405 8786297

[B141] Munoz-Fernandez R. Blanco F. J. Frecha C. Martin F. Kimatrai M. Abadia-Molina A. C. Garcia-Pacheco J. M. Olivares E. G. (2006). Follicular dendritic cells are related to bone marrow stromal cell progenitors and to myofibroblasts. J. Immunol. 177, 280–289 16785523 10.4049/jimmunol.177.1.280

[B142] Murakami T. Chen X. Hase K. Sakamoto A. Nishigaki C. Ohno H. (2007). Splenic CD19-CD35+B220+ cells function as an inducer of follicular dendritic cell network formation. Blood 110, 1215–1224 10.1182/blood-2007-01-068387 17519390 PMC1939903

[B143] Natkunam Y. (2007). The biology of the germinal center. Hematology Am. Soc. Hematol. Educ. Program. 2007, 210–215 10.1182/asheducation-2007.1.210 18024632

[B144] Nieuwenhuis P. Opstelten D. (1984). Functional anatomy of germinal centers. Am. J. Anat. 170, 421–435 10.1002/aja.1001700315 6383007

[B145] Nossal G. J. Abbot A. Mitchell J. Lummus Z. (1968). Antigens in immunity. XV. Ultrastructural features of antigen capture in primary and secondary lymphoid follicles. J. Exp. Med. 127, 277–290 4169585 10.1084/jem.127.2.277 PMC2138444

[B146] Nutt S. L. Tarlinton D. M. (2011). Germinal center B and follicular helper T cells: siblings, cousins or just good friends? Nat. Immunol. 12, 472–477 21739669 10.1038/ni.2019

[B147] Nuvolone M. Aguzzi A. Heikenwalder M. (2009). Cells and prions: a license to replicate. FEBS Lett. 583, 2674–2684 10.1016/j.febslet.2009.06.014 19527722

[B148] Ok C. Y. Singh R. R. Vega F. (2012). Aberrant activation of the hedgehog signaling pathway in malignant hematological neoplasms. Am. J. Pathol. 180, 2–11 10.1016/j.ajpath.2011.09.009 22056910 PMC3338341

[B149] Or-Guil M. Wittenbrink N. Weiser A. A. Schuchhardt J. (2007). Recirculation of germinal center B cells: a multilevel selection strategy for antibody maturation. Immunol. Rev. 216, 130–141 10.1111/j.1600-065X.2007.00507.x 17367339

[B150] Park C. S. Yoon S. O. Armitage R. J. Choi Y. S. (2004). Follicular dendritic cells produce IL-15 that enhances germinal center B cell proliferation in membrane-bound form. J. Immunol. 173, 6676–6683 15557159 10.4049/jimmunol.173.11.6676

[B151] Parker D. C. (1993). T cell-dependent B cell activation. Annu. Rev. Immunol. 11, 331–360 10.1146/annurev.iy.11.040193.001555 8476565

[B152] Payet-Jamroz M. Helm S. L. Wu J. Kilmon M. Fakher M. Basalp A. Tew J. G. Szakal A. K. Noben-Trauth N. Conrad D. H. (2001). Suppression of IgE responses in CD23-transgenic animals is due to expression of CD23 on nonlymphoid cells. J. Immunol. 166, 4863–4869 11290762 10.4049/jimmunol.166.8.4863

[B153] Peperzak V. Vikstrom I. B. Tarlinton D. M. (2012). Through a glass less darkly: apoptosis and the germinal center response to antigen. Immunol. Rev. 247, 93–106 10.1111/j.1600-065X.2012.01123.x 22500834

[B154] Qin D. Wu J. Burton G. F. Szakal A. K. Tew J. G. (1997). A role for CR2 in FDC-B cell interactions. Adv. Exp. Med. Biol. 417, 493–497 9286409 10.1007/978-1-4757-9966-8_81

[B155] Qin D. Wu J. Carroll M. C. Burton G. F. Szakal A. K. Tew J. G. (1998). Evidence for an important interaction between a complement-derived CD21 ligand on follicular dendritic cells and CD21 on B cells in the initiation of IgG responses. J. Immunol. 161, 4549–4554 9794381

[B156] Qin D. Wu J. Vora K. A. Ravetch J. V. Szakal A. K. Manser T. Tew J. G. (2000). Fc gamma receptor IIB on follicular dendritic cells regulates the B cell recall response. J. Immunol. 164, 6268–6275 10843680 10.4049/jimmunol.164.12.6268

[B157] Rahman Z. S. (2011). Impaired clearance of apoptotic cells in germinal centers: implications for loss of B cell tolerance and induction of autoimmunity. Immunol. Res. 51, 125–133 10.1007/s12026-011-8248-4 22038528

[B158] Reparon-Schuijt C. C. Van Esch W. J. Van Kooten C. Schellekens G. A. de Jong B. A. van Venrooij W. J. Breedveld F. C. Verweij C. L. (2001). Secretion of anti-citrulline-containing peptide antibody by B lymphocytes in rheumatoid arthritis. Arthritis Rheum. 44, 41–47 10.1002/1529-0131(200101)44:1<41::AID-ANR6>3.0.CO;2-0 11212174

[B159] Rohn T. A. Jennings G. T. Hernandez M. Grest P. Beck M. Zou Y. Kopf M. Bachmann M. F. (2006). Vaccination against IL-17 suppresses autoimmune arthritis and encephalomyelitis. Eur. J. Immunol. 36, 2857–2867 10.1002/eji.200636658 17048275

[B160] Sacedon R. Diez B. Nunez V. Hernandez-Lopez C. Gutierrez-Frias C. Cejalvo T. Outram S. V. Crompton T. Zapata A. G. Vicente A. Varas A. (2005). Sonic hedgehog is produced by follicular dendritic cells and protects germinal center B cells from apoptosis. J. Immunol. 174, 1456–1461 15661904 10.4049/jimmunol.174.3.1456

[B161] Sauerborn M. Brinks V. Jiskoot W. Schellekens H. (2010). Immunological mechanism underlying the immune response to recombinant human protein therapeutics. Trends Pharmacol. Sci. 31, 53–59 10.1016/j.tips.2009.11.001 19963283

[B162] Schneider P. (2005). The role of APRIL and BAFF in lymphocyte activation. Curr. Opin. Immunol. 17, 282–289 10.1016/j.coi.2005.04.005 15886118

[B163] Schnizlein C. T. Kosco M. H. Szakal A. K. Tew J. G. (1985). Follicular dendritic cells in suspension: identification, enrichment, and initial characterization indicating immune complex trapping and lack of adherence and phagocytic activity. J. Immunol. 134, 1360–1368 3968423

[B164] Schwickert T. A. Lindquist R. L. Shakhar G. Livshits G. Skokos D. Kosco-Vilbois M. H. Dustin M. L. Nussenzweig M. C. (2007). *In vivo* imaging of germinal centres reveals a dynamic open structure. Nature 446, 83–87 10.1038/nature05573 17268470

[B165] Sfriso P. Ghirardello A. Botsios C. Tonon M. Zen M. Bassi N. Bassetto F. Doria A. (2010). Infections and autoimmunity: the multifaceted relationship. J. Leukoc. Biol. 87, 385–395 10.1189/jlb.0709517 20015961

[B166] Shlomchik M. J. Weisel F. (2012a). Germinal center selection and the development of memory B and plasma cells. Immunol. Rev. 247, 52–63 10.1111/j.1600-065X.2012.01124.x 22500831

[B167] Shlomchik M. J. Weisel F. (2012b). Germinal centers. Immunol. Rev. 247, 5–10 10.1111/j.1600-065X.2012.01125.x 22500827

[B168] Shulga-Morskaya S. Dobles M. Walsh M. E. Ng L. G. Mackay F. Rao S. P. Kalled S. L. Scott M. L. (2004). B cell-activating factor belonging to the TNF family acts through separate receptors to support B cell survival and T cell-independent antibody formation. J. Immunol. 173, 2331–2341 15294946 10.4049/jimmunol.173.4.2331

[B169] Sipos F. Muzes G. (2011). Isolated lymphoid follicles in colon: switch points between inflammation and colorectal cancer? World J. Gastroenterol. 17, 1666–1673 10.3748/wjg.v17.i13.1666 21483625 PMC3072629

[B170] Smith B. A. Gartner S. Liu Y. Perelson A. S. Stilianakis N. I. Keele B. F. Kerkering T. M. Ferreira-Gonzalez A. Szakal A. K. Tew J. G. Burton G. F. (2001). Persistence of infectious HIV on follicular dendritic cells. J. Immunol. 166, 690–696 11123354 10.4049/jimmunol.166.1.690

[B171] Smith J. P. Burton G. F. Tew J. G. Szakal A. K. (1998). Tingible body macrophages in regulation of germinal center reactions. Dev. Immunol. 6, 285–294 9814602 10.1155/1998/38923 PMC2276033

[B172] Smith J. P. Lister A. M. Tew J. G. Szakal A. K. (1991). Kinetics of the tingible body macrophage response in mouse germinal center development and its depression with age. Anat. Rec. 229, 511–520 10.1002/ar.1092290412 2048755

[B173] Smith K. G. Clatworthy M. R. (2010). FcgammaRIIB in autoimmunity and infection: evolutionary and therapeutic implications. Nat. Rev. Immunol. 10, 328–343 10.1038/nri2762 20414206 PMC4148599

[B174] Smith-Franklin B. A. Keele B. F. Tew J. G. Gartner S. Szakal A. K. Estes J. D. Thacker T. C. Burton G. F. (2002). Follicular dendritic cells and the persistence of HIV infectivity: the role of antibodies and Fcgamma receptors. J. Immunol. 168, 2408–2414 11859132 10.4049/jimmunol.168.5.2408

[B175] Sonderegger I. Rohn T. A. Kurrer M. O. Iezzi G. Zou Y. Kastelein R. A. Bachmann M. F. Kopf M. (2006). Neutralization of IL-17 by active vaccination inhibits IL-23-dependent autoimmune myocarditis. Eur. J. Immunol. 36, 2849–2856 10.1002/eji.200636484 17039570

[B176] Song H. Nie X. Basu S. Cerny J. (1998). Antibody feedback and somatic mutation in B cells: regulation of mutation by immune complexes with IgG antibody. Immunol. Rev. 162, 211–218 9602366 10.1111/j.1600-065x.1998.tb01443.x

[B177] Spohn G. Guler R. Johansen P. Keller I. Jacobs M. Beck M. Rohner F. Bauer M. Dietmeier K. Kundig T. M. Jennings G. T. Brombacher F. Bachmann M. F. (2007). A virus-like particle-based vaccine selectively targeting soluble TNF-alpha protects from arthritis without inducing reactivation of latent tuberculosis. J. Immunol. 178, 7450–7457 17513796 10.4049/jimmunol.178.11.7450

[B178] Spohn G. Keller I. Beck M. Grest P. Jennings G. T. Bachmann M. F. (2008). Active immunization with IL-1 displayed on virus-like particles protects from autoimmune arthritis. Eur. J. Immunol. 38, 877–887 10.1002/eji.200737989 18253928

[B179] Spohn G. Schwarz K. Maurer P. Illges H. Rajasekaran N. Choi Y. Jennings G. T. Bachmann M. F. (2005). Protection against osteoporosis by active immunization with TRANCE/RANKL displayed on virus-like particles. J. Immunol. 175, 6211–6218 16237119 10.4049/jimmunol.175.9.6211

[B180] Sukumar S. Conrad D. H. Szakal A. K. Tew J. G. (2006a). Differential T cell-mediated regulation of CD23 (Fc epsilonRII) in B cells and follicular dendritic cells. J. Immunol. 176, 4811–4817 16585575 10.4049/jimmunol.176.8.4811

[B181] Sukumar S. Szakal A. K. Tew J. G. (2006b). Isolation of functionally active murine follicular dendritic cells. J. Immunol. Methods 313, 81–95 10.1016/j.jim.2006.03.018 16824539

[B182] Sukumar S. El Shikh M. E. Tew J. G. Szakal A. K. (2008). Ultrastructural study of highly enriched follicular dendritic cells reveals their morphology and the periodicity of immune complex binding. Cell Tissue Res. 332, 89–99 10.1007/s00441-007-0566-4 18236080

[B183] Sulzer B. Perelson A. S. (1997). Immunons revisited: binding of multivalent antigens to B cells. Mol. Immunol. 34, 63–74 10.1016/S0161-5890(96)00096-X 9182877

[B184] Suzuki K. Grigorova I. Phan T. G. Kelly L. M. Cyster J. G. (2009). Visualizing B cell capture of cognate antigen from follicular dendritic cells. J. Exp. Med. 206, 1485–1493 10.1016/j.healun.2008.01.012 19506051 PMC2715076

[B185] Suzuki K. Maruya M. Kawamoto S. Sitnik K. Kitamura H. Agace W. W. Fagarasan S. (2010). The sensing of environmental stimuli by follicular dendritic cells promotes immunoglobulin A generation in the gut. Immunity 33, 71–83 10.1016/j.immuni.2010.07.003 20643338

[B186] Szakal A. K. Aydar Y. Balogh P. Tew J. G. (2002). Molecular interactions of FDCs with B cells in aging. Semin. Immunol. 14, 267–274 10.1016/S1044-5323(02)00059-3 12163302

[B187] Szakal A. K. Gieringer R. L. Kosco M. H. Tew J. G. (1985). Isolated follicular dendritic cells: cytochemical antigen localization, Nomarski, SEM, and TEM morphology. J. Immunol. 134, 1349–1359 3881522

[B188] Szakal A. K. Hanna M. G. Jr. (1968). The ultrastructure of antigen localization and viruslike particles in mouse spleen germinal centers. Exp. Mol. Pathol. 8, 75–89 10.1016/0014-4800(68)90007-5 4170142

[B189] Szakal A. K. Holmes K. L. Tew J. G. (1983). Transport of immune complexes from the subcapsular sinus to lymph node follicles on the surface of nonphagocytic cells, including cells with dendritic morphology. J. Immunol. 131, 1714–1727 6619542

[B190] Szakal A. K. Kapasi Z. F. Haley S. T. Tew J. G. (1995a). A theory of follicular dendritic cell origin. Curr. Top. Microbiol. Immunol. 201, 1–13 7587345 10.1007/978-3-642-79603-6_1

[B191] Szakal A. K. Kapasi Z. F. Haley S. T. Tew J. G. (1995b). Multiple lines of evidence favoring a bone marrow derivation of follicular dendritic cells (FDCs). Adv. Exp. Med. Biol. 378, 267–272 8526070 10.1007/978-1-4615-1971-3_59

[B192] Szakal A. K. Kapasi Z. F. Masuda A. Tew J. G. (1992). Follicular dendritic cells in the alternative antigen transport pathway: microenvironment, cellular events, age and retrovirus related alterations. Semin. Immunol. 4, 257–265 1382662

[B193] Szakal A. K. Kosco M. H. Tew J. G. (1988). A novel *in vivo* follicular dendritic cell-dependent iccosome-mediated mechanism for delivery of antigen to antigen-processing cells. J. Immunol. 140, 341–353 3257233

[B194] Szakal A. K. Taylor J. K. Smith J. P. Kosco M. H. Burton G. F. Tew J. J. (1990). Kinetics of germinal center development in lymph nodes of young and aging immune mice. Anat. Rec. 227, 475–485 10.1002/ar.1092270411 2393099

[B195] Szomolanyi-Tsuda E. Welsh R. M. (1998). T-cell-independent antiviral antibody responses. Curr. Opin. Immunol. 10, 431–435 10.1016/S0952-7915(98)80117-9 9722919

[B196] Tan J. K. Watanabe T. (2010). Artificial engineering of secondary lymphoid organs. Adv. Immunol. 105, 131–157 10.1016/S0065-2776(10)05005-4 20510732

[B197] Tarlinton D. (1998). Germinal centers: form and function. Curr. Opin. Immunol. 10, 245–251 10.1016/S0952-7915(98)80161-1 9638359

[B198] Tarlinton D. M. (2008). Evolution in miniature: selection, survival and distribution of antigen reactive cells in the germinal centre. Immunol. Cell Biol. 86, 133–138 10.1038/sj.icb.7100148 18180800

[B199] Taylor P. R. Pickering M. C. Kosco-Vilbois M. H. Walport M. J. Botto M. Gordon S. Martinez-Pomares L. (2002). The follicular dendritic cell restricted epitope, FDC-M2, is complement C4; localization of immune complexes in mouse tissues. Eur. J. Immunol. 32, 1888–1896 10.1002/1521-4141(200207)32:7<1883::AID-IMMU1888>3.0.CO;2-8 12115608

[B200] Tew J. G. Burton G. F. Kupp L. I. Szakal A. (1993). Follicular dendritic cells in germinal center reactions. Adv. Exp. Med. Biol. 329, 461–465 8379410 10.1007/978-1-4615-2930-9_77

[B201] Tew J. G. Greene E. J. Makoski M. H. (1976). *In vitro* evidence indicating a role for the Fc region of IgG in the mechanism for the long-term maintenance and regulation of antibody levels *in vivo*. Cell. Immunol. 26, 141–152 975248 10.1016/0008-8749(76)90358-0

[B202] Tew J. G. Mandel T. E. Miller G. A. (1979). Immune retention: immunological requirements for maintaining an easily degradable antigen *in vivo*. Aust. J. Exp. Biol. Med. Sci. 57, 401–414 94545 10.1038/icb.1979.40

[B203] Tew J. G. Phipps R. P. Mandel T. E. (1980). The maintenance and regulation of the humoral immune response: persisting antigen and the role of follicular antigen-binding dendritic cells as accessory cells. Immunol. Rev. 53, 175–201 7009404 10.1111/j.1600-065x.1980.tb01044.x

[B204] Tew J. G. Thorbecke G. J. Steinman R. M. (1982). Dendritic cells in the immune response: characteristics and recommended nomenclature (A report from the Reticuloendothelial Society Committee on Nomenclature). J. Reticuloendothel. Soc. 31, 371–380 6750114

[B205] Tew J. G. Wu J. Fakher M. Szakal A. K. Qin D. (2001). Follicular dendritic cells: beyond the necessity of T-cell help. Trends Immunol. 22, 361–367 10.1016/S1471-4906(01)01942-1 11429319

[B206] Thacker T. C. Zhou X. Estes J. D. Jiang Y. Keele B. F. Elton T. S. Burton G. F. (2009). Follicular dendritic cells and human immunodeficiency virus type 1 transcription in CD4+ T cells. J. Virol. 83, 150–158 10.1128/JVI.01652-08 18971284 PMC2612309

[B207] Thiel J. Kimmig L. Salzer U. Grudzien M. Lebrecht D. Hagena T. Draeger R. Volxen N. Bergbreiter A. Jennings S. Gutenberger S. Aichem A. Illges H. Hannan J. P. Kienzler A. K. Rizzi M. Eibel H. Peter H. H. Warnatz K. Grimbacher B. Rump J. A. Schlesier M. (2012). Genetic CD21 deficiency is associated with hypogammaglobulinemia. J. Allergy Clin. Immunol. 129, 801–810 10.1016/j.jaci.2011.09.027 22035880

[B208] Thorbecke G. J. Amin A. R. Tsiagbe V. K. (1994). Biology of germinal centers in lymphoid tissue. FASEB J. 8, 832–840 8070632 10.1096/fasebj.8.11.8070632

[B209] Tumanov A. V. Grivennikov S. I. Kruglov A. A. Shebzukhov Y. V. Koroleva E. P. Piao Y. Cui C. Y. Kuprash D. V. Nedospasov S. A. (2010). Cellular source and molecular form of TNF specify its distinct functions in organization of secondary lymphoid organs. Blood 116, 3456–3464 10.1182/blood-2009-10-249177 20634375 PMC3321833

[B210] Usui K. Honda S. Yoshizawa Y. Nakahashi-Oda C. Tahara-Hanaoka S. Shibuya K. Shibuya A. (2012). Isolation and characterization of naive follicular dendritic cells. Mol. Immunol. 50, 172–176 10.1016/j.molimm.2011.11.010 22189408

[B211] Varin M. M. Le Pottier L. Youinou P. Saulep D. Mackay F. Pers J. O. (2010). B-cell tolerance breakdown in Sjogren's syndrome: focus on BAFF. Autoimmun. Rev. 9, 604–608 10.1016/j.autrev.2010.05.006 20457281

[B212] Victoratos P. Kollias G. (2009). Induction of autoantibody-mediated spontaneous arthritis critically depends on follicular dendritic cells. Immunity 30, 130–142 10.1016/j.immuni.2008.10.019 19119026

[B213] Vinuesa C. G. Cyster J. G. (2011). How T cells earn the follicular rite of passage. Immunity 35, 671–680 10.1016/j.immuni.2011.11.001 22118524

[B214] Vinuesa C. G. Linterman M. A. Goodnow C. C. Randall K. L. (2010). T cells and follicular dendritic cells in germinal center B-cell formation and selection. Immunol. Rev. 237, 72–89 10.1111/j.1600-065X.2010.00937.x 20727030

[B215] Vinuesa C. G. Sanz I. Cook M. C. (2009). Dysregulation of germinal centres in autoimmune disease. Nat. Rev. Immunol. 9, 845–857 10.1038/nri2637 19935804

[B216] Vos Q. Lees A. Wu Z. Q. Snapper C. M. Mond J. J. (2000). B-cell activation by T-cell-independent type 2 antigens as an integral part of the humoral immune response to pathogenic microorganisms. Immunol. Rev. 176, 154–170 10.1034/j.1600-065X.2000.00607.x 11043775

[B216a] Wang X. Cho B. Suzuki K. Xu Y. Green J. A. An J. Cyster J. G. (2011). Follicular dendritic cells help establish follicle identity and promote B cell retention in germinal centers. J. Exp. Med. 208, 2497–2510 10.1084/jem.20111449 22042977 PMC3256970

[B217] Weinstein J. S. Hernandez S. G. Craft J. (2012). T cells that promote B-Cell maturation in systemic autoimmunity. Immunol. Rev. 247, 160–171 10.1111/j.1600-065X.2012.01122.x 22500839 PMC3334351

[B218] Wen L. Shinton S. A. Hardy R. R. Hayakawa K. (2005). Association of B-1 B cells with follicular dendritic cells in spleen. J. Immunol. 174, 6918–6926 15905534 10.4049/jimmunol.174.11.6918

[B219] Wilke G. Steinhauser G. Grun J. Berek C. (2010). In silico subtraction approach reveals a close lineage relationship between follicular dendritic cells and BP3(hi) stromal cells isolated from SCID mice. Eur. J. Immunol. 40, 2165–2173 10.1002/eji.200940202 20518031

[B220] Wittenbrink N. Klein A. Weiser A. A. Schuchhardt J. Or-Guil M. (2011). Is there a typical germinal center? A large-scale immunohistological study on the cellular composition of germinal centers during the hapten-carrier-driven primary immune response in mice. J. Immunol. 187, 6185–6196 10.4049/jimmunol.1101440 22102720

[B221] Wu Y. El Shikh M. E. El Sayed R. M. Best A. M. Szakal A. K. Tew J. G. (2009). IL-6 produced by immune complex-activated follicular dendritic cells promotes germinal center reactions, IgG responses and somatic hypermutation. Int. Immunol. 21, 745–756 10.1093/intimm/dxp041 19461124 PMC2686616

[B222] Wu Y. Sukumar S. El Shikh M. E. Best A. M. Szakal A. K. Tew J. G. (2008). Immune complex-bearing follicular dendritic cells deliver a late antigenic signal that promotes somatic hypermutation. J. Immunol. 180, 281–290 18097029 10.4049/jimmunol.180.1.281

[B223] Yoon S. O. Zhang X. Berner P. Blom B. Choi Y. S. (2009). Notch ligands expressed by follicular dendritic cells protect germinal center B cells from apoptosis. J. Immunol. 183, 352–358 10.4049/jimmunol.0803183 19542446

[B224] Zhang X. Li L. Jung J. Xiang S. Hollmann C. Choi Y. S. (2001). The distinct roles of T cell-derived cytokines and a novel follicular dendritic cell-signaling molecule 8D6 in germinal center-B cell differentiation. J. Immunol. 167, 49–56 11418631 10.4049/jimmunol.167.1.49

[B225] Zhang X. Park C. S. Yoon S. O. Li L. Hsu Y. M. Ambrose C. Choi Y. S. (2005). BAFF supports human B cell differentiation in the lymphoid follicles through distinct receptors. Int. Immunol. 17, 779–788 10.1093/intimm/dxh259 15908449

[B226] Zheng B. Han S. Takahashi Y. Kelsoe G. (1997). Immunosenescence and germinal center reaction. Immunol. Rev. 160, 63–77 9476666 10.1111/j.1600-065x.1997.tb01028.x

[B227] Zubler R. H. (2001). Naive and memory B cells in T-cell-dependent and T-independent responses. Springer Semin. Immunopathol. 23, 405–419 11826617 10.1007/s281-001-8167-7

